# Comparative transcriptome expression analysis in susceptible and resistant potato (*Solanum tuberosum*) cultivars to common scab (*Streptomyces scabies*) revealed immune priming responses in the incompatible interaction

**DOI:** 10.1371/journal.pone.0235018

**Published:** 2020-07-16

**Authors:** Bourlaye Fofana, Ashok Somalraju, Sherry Fillmore, Mohsin Zaidi, David Main, Kaushik Ghose

**Affiliations:** 1 Charlottetown Research and Development Centre, Agriculture and Agri-Food Canada, Charlottetown, Prince Edward Island, Canada; 2 Kentville Research and Development Centre, Agriculture and Agri-Food Canada, Kentville, Nova Scotia, Canada; 3 Department of Plant and Soil Science, Texas Tech University, Lubbock, Texas, United States of America; Universite Paris-Sud, FRANCE

## Abstract

Common scab disease in potato has become a widespread issue in major potato production areas, leading to increasing economic losses. Varietal resistance is seen as a viable and long-term scab management strategy. However, the genes and mechanisms of varietal resistance are unknown. In the current study, a comparative RNA transcriptome sequencing and differential gene signaling and priming sensitization studies were conducted in two potato cultivars that differ by their response to common scab (*Streptomyces scabies)*, for unraveling the genes and pathways potentially involved in resistance within this pathosystem. We report on a consistent and contrasted gene expression pattern from 1,064 annotated genes differentiating a resistant (Hindenburg) and a susceptible (Green Mountain) cultivars, and identified a set of 273 co-regulated differentially expressed genes in 34 pathways that more likely reflect the genetic differences of the cultivars and metabolic mechanisms involved in the scab pathogenesis and resistance. The data suggest that comparative transcriptomic phenotyping can be used to predict scab lesion phenotype in breeding lines using mature potato tuber. The study also showed that the resistant cultivar, Hindenburg, has developed and maintained a capacity to sense and prime itself for persistent response to scab disease over time, and suggests an immune priming reaction as a mechanism for induced-resistance in scab resistant potato cultivars. The set of genes identified, described, and discussed in the study paves the foundation for detailed characterizations towards tailoring and designing procedures for targeted gene knockout through gene editing and phenotypic evaluation.

## Introduction

Common scab (*Streptomyces scabies)* disease in potato has become a widespread issue in the major potato production areas in Canada, leading to increased economic losses [1]. Eighty two percent (82%) of the farmers surveyed in Canada in 2003 were found to be experiencing common scab problems on their farm, with an estimated economic loss of 15 to 17 million dollars [2]. Since then, despite application of integrated agronomic and cultural practices including soil pH and moisture control, crop rotation, seed treatment, and the use of tolerant cultivars when available [3–5], scab incidence is still rising in most production areas (Robert Coffin, 2017, personal communication). Among scab control methods, varietal resistance to common scab has always been regarded as one of the most sustainable and environmentally friendly options [6,7]. In this context, the less commercially successful German potato cultivar Hindenburg (HB) that expresses a high level of resistance to common scab [8] has been used as a scab resistance source in many breeding programs [6,9]. It has been suggested that scab resistance in potato is a quantitative polygenic trait, controlled by a small number of genes [9,10], which are currently unknown.

As symptoms, common scab causes superficial, erumpent or deep-pitted lesions to the tuber skin following the bacterial entry through the lenticels or by direct penetration through the immature young tuber periderm [5,11]. The pathogen causes cell hypertrophy, cell collapse, and death during active expansion of young plant tissue [12] by the action of thaxtomin A, a secreted bacterial toxin and the key virulent factor responsible for the inhibition of cellulose biosynthesis [13,14]. To overcome the plant’s defense system, phyto-pathogens such as *Pseumodonas syringae* and *Streptomyces scabies* have evolved strategies to manipulate the plant hormone signalling pathways, making them vulnerable for successful infection. Hence, they activate the plant jasmonate pathway to deactivate the salicylic acid (SA) pathway which is involved in the plant defense signaling against biotrophic and hemibiotrophic pathogens [15,16]. After successful infection, scab bacterium uses suberin as source of carbon [17,18]. In response to the bacterial infection and periderm wounding, tissue around the lesion divide rapidly, form a suberized wall layer to heal the infected portions, leaving suberized raised corky cells or pitted-wound tissue layer sealing off the wound at the tuber surface [19]. Suberin is composed of poly-aliphatic and poly-aromatic compounds linked to a lignin-like structure by esterification to ferulic acid [20], and the differences in the suberin phenolic composition as well as the level of suberization have been found to be associated with scab resistance [17]. However, the genes and pathways involved in such relationships are not well understood. Whereas recent studies focusing on differential gene expression have been reported in scab tolerant and susceptible potato clones at early stage of the tuberization [21], in suberin biosynthesis [5], and during the healing process of wound-induced suberization [22], the genetic basis and putative mechanisms of potato cultivar susceptibility and resistance to common scab are not well established and well known. Moreover, the question as to why resistant cultivars display less infection and very few lesions when the susceptible clones are fully infected and carry many deep-pitted lesions remains so far unanswered. Upon attack by necrotizing pathogens or treatment with natural compounds such as salicylic acid or β-aminobyturic acid (ABA), many plants develop an enhanced capacity for activating stronger defense responses by mobilizing infection-induced cellular defense responses, a process called priming [23–27]. Thus, plant pathogen recognition and immune priming have been proposed as a memory-based mechanism for induced-resistance in plants [28], and induced expression of genes including *mitogen-activated protein kinases* (*MPKs*) [29,30], *Subtilisin* [28] and *enhanced disease resistance 1* (*EDR1*) have been associated with priming in plants [23,31–33]. It has been shown that mutation of *EDR1* leads to resistance phenotypes, induces callose deposition, and appears to function through salicylic acid-dependent pathways while being inducible by pathogen attacks and elicitors [34]. Nonetheless, it is not documented whether such a mechanism exists in the potato-scab pathosystem and if *MPKs*, *Subtilisin* and *EDR1* are inducible following scab infection. We hypothesized that scab resistant potato cultivars might have developed and kept such a robust priming phenomenon throughout the tuber phenology and thus, may display differential signaling and priming response for resistance when compared to its susceptible counterpart at any stage of their development.

Thus, the current study was undertaken to test this hypothesis, and in particular to 1) investigate whether comparative differential gene expression profile of mature and immature tubers from scab resistant and susceptible cultivars correlates the observed differential phenotypic reactions to common scab in the field; 2) identify key co-regulated pathways and genes potentially contributing to, or correlated with disease resistance and which may be of interest for scab breeding; and 3) evaluate potential priming genes in this pathosystem. To address these fundamental questions, an RNA transcriptomic sequencing approach was used and was complemented with pathogen-induced signalling studies. Here, we report on a set of genes differentially expressed in mature tuber of the scab-resistant potato cultivar HB and the susceptible cultivar Green Mountain (GM) as potential targets for further functional analysis, some of which have previously been reported in a resistant cultivar during the infection process at the early stage of tuberization [21]. Furthermore, we show that HB has developed and maintain a capacity to sense and prime itself for persistent response to scab disease over time, and we suggest an induced immune priming reaction as a mechanism for induced-resistance in this scab resistant potato cultivar.

## Methods

### Plant materials

#### Field experiments

**Fig 1:**
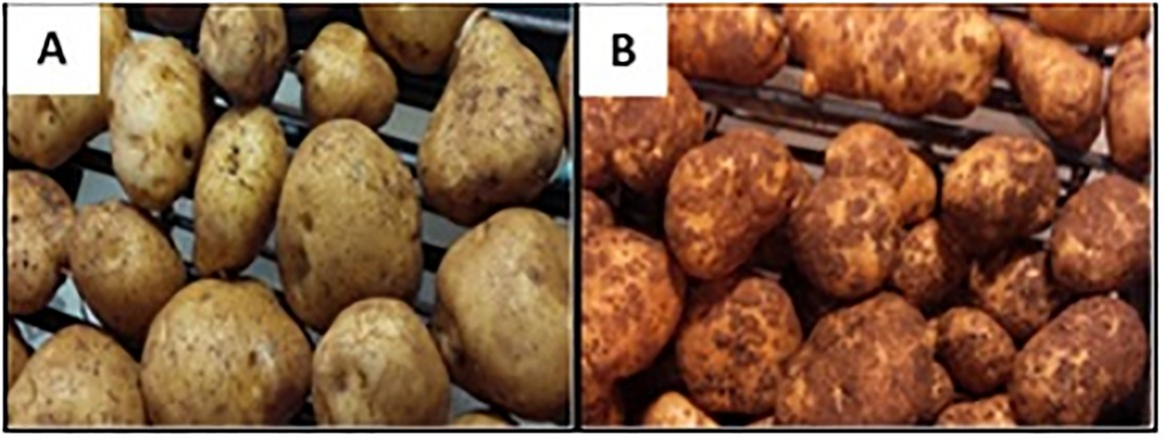
Photographs showing common scab phenotypic reactions. **A**, the scab-resistant cultivar Hindenburg (HB); and **B**, the scab susceptible cultivar Green Mountain (GM). More scab lesions can be seen in the forms of pitted-lesions or necrosis on the skin of GM.

The plant materials used for the RNAseq transcriptomic study consisted of mature potato (*Solanum tuberosum* L) tubers collected from the late maturing potato cultivars HB (parentage: Ismene x Jubel) and GM grown in a field artificially infested with common scab by dumping scabby potato culls overs years. HB is a highly scab-resistant cultivar whereas GM is highly susceptible [8,35]. Thus, and HB has been used as scab resistance source in many breeding programs [9]. Briefly, the certified seed potatoes from each cultivar were obtained from the AAFC Fredericton Research and Development Centre (NB, Canada). Tubers were planted and grown in the same field that is under an heavy artificial epiphytotic scab pressure and used as part of the Agriculture and Agri-Food Canada’s national scab evaluation trials at the Harrington Research Farm (PE, Canada) in the summer of 2016. HB and GM always served as resistant and susceptible checks, respectively during these national scab trials. Each plot was planted with 30 seed potatoes, randomized in 4 replicates across the field, and grown under conventional agronomic practices. At harvest, mature tubers were collected separately from each plot and stored at 4°C for 2 weeks. Tubers were then, washed, processed, and graded for scab reactions according to the Canadian Food Inspection Agency’s fresh fruit or vegetable grade requirement (Fig 1). A total of ten tubers per replicate for each cultivar were taken to the laboratory for a later tissue collection and RNA extraction.

#### Greenhouse experiments

**Fig 2:**
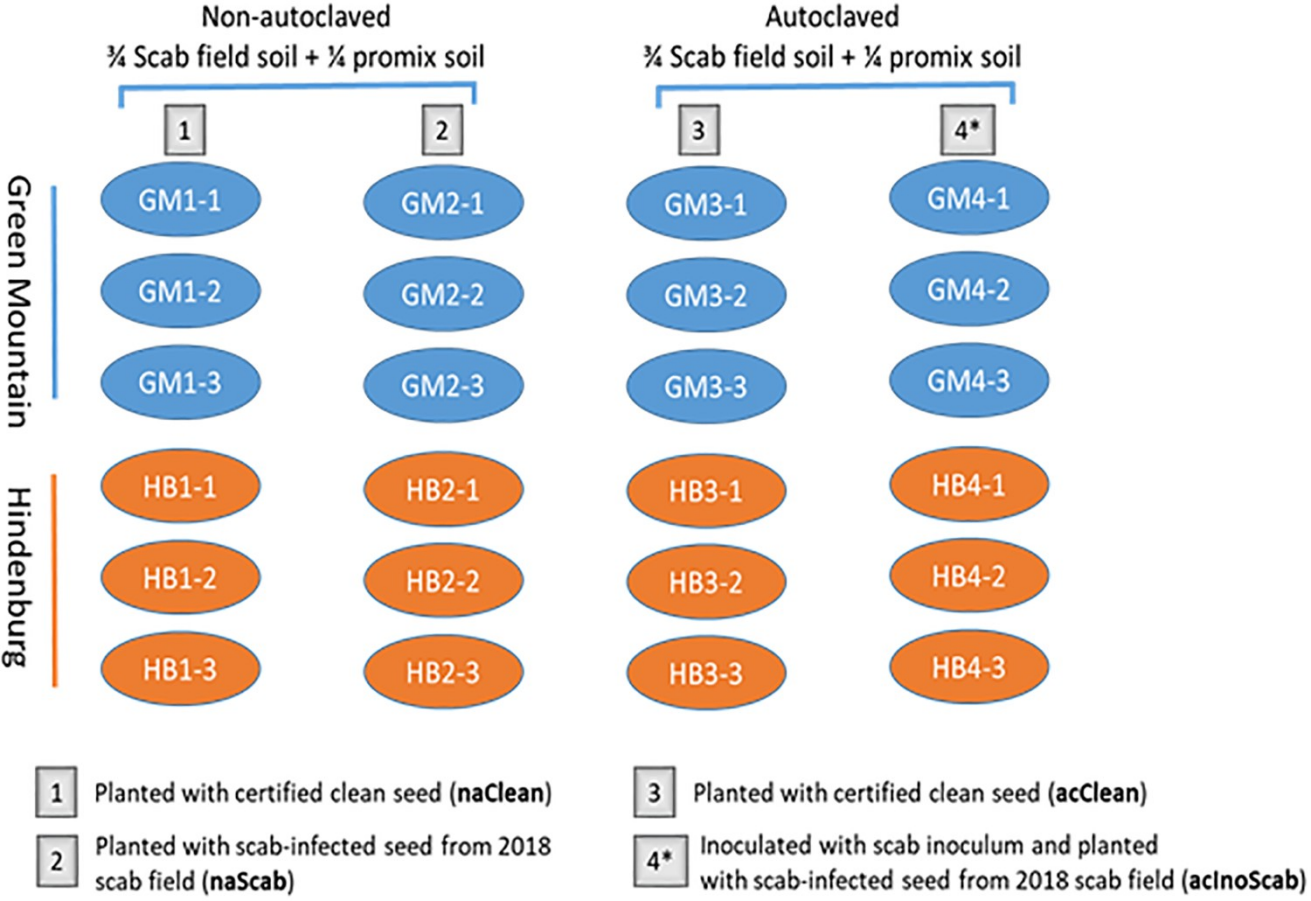
Experimental layout for scab infection trial under controlled environment. Planting conditions and treatments are indicated by number 1–4. Treatment 1: naClean, non-autoclaved soil planted with certified clean seed; Treatment 2: naScab, non-autoclaved soil planted with scab-infected seed from the 2018 infected field. Treatment 3: acClean, autoclaved soil planted with certified clean seed; Treatment 4: acInoScab, autoclaved soil inoculated with scab inoculum and planted with scab-infected seed from the 2018 infected field. Each cultivar (GM and HB) was grown in three replicates in each treatment.

To further ascertain the scab symptoms observed under the field conditions as due to natural scab infection rather than other pathogens, and to provide more insights into potential transgenerational priming (a phenomenon referred to as a priming status inherited by offspring of previously primed plants) effects [26,27] in the two cultivars, plants were grown in a greenhouse during the winter (February) 2019. The plant growth conditions involved three soil types including a non-sterile (non-autoclaved) 2018 scab field soil (na), a sterile (autoclaved) 2018 scab field soil (ac), and a sterile (autoclaved) 2018 scab field soil inoculated with scab inoculum (acIno). The scab inoculum was obtained by peeling the corky skin of GM. The skin was dried at 37°C for 24 h and reduced to fine powder using a KitchenAid blinder (St Joseph, MI, USA), and 10 g of skin powder was mixed with the sterile 2018 scab field soil of each individual pot used for this soil condition. Two kinds of potato seed sets were used for planting: one type of seed set consisted of scab-infected seeds (Scab) of each cultivar harvested from the 2018 scab field trial, and the other set consisted of clean certified seeds obtained from the AAFC Fredericton Research and Development Centre (Clean) as for those planted in the field trial. The three types of soils were planted with the two kinds of seeds, totalling four treatments referred to as treatment 1 (naClean), 2 (naScab), 3 (acClean), and 4 (acInoScab). Each cultivar was planted as triplicated pots in each of the four treatments (Fig 2). Pots were adequately watered (50 mL, twice/day) using an automatic watering system (Senninger Irrigation Inc.) and fertilized as required. Plants were maintained under 8/16 h photoperiod at 22°C from February to Spring (June) 2019.

At the first flowering stage, which coincides with tuber initiation, 1–2 mini tubers were collected from each treatment. This first sampling was referred to as time point 1, and the plants were left to grow until bulking and maturity. Three pots (1 pot with GM and 2 pots with HB) had not yet produced tubers at this first sampling time point. Thus, only 21 collected mini tuber samples were rinsed with distilled water and the scab symptoms were rated as previously described, then photographed, diced, flash-frozen in liquid nitrogen, and stored at -80°C until further use in RNA extractions. After 110 days post planting, plants were subjected to water restriction for two weeks, with water supplied every two days until full senescence in June 2019, approximatively 120–130 days after planting. At maturity, tubers were collected from all 24 pots and referred to as time point 2. The tubers were washed, photographed. The scab symptoms and incidence were recorded, and tubers processed and stored at -80°C as described for the mini tubers.

### Tissue sampling, total RNA extraction and quality control

For the transcriptomic studies, three of the four biological replicates in the field trial were used for RNA extraction. One tuber showing evidence of scab lesions was selected from each replicate of HB or GM and considered as a biological unit. Hence, a total of three tubers representing the three replicates per cultivar was processed for RNA extraction. The tubers were further washed with water and cleaned with 70% ethanol before tissue collection. For each tuber, four tissue samples consisting of 1.5–2 mm thick skin cores pealed from four locations were collected following previous tissue collection method [36]. The four tissue samples from each tuber were pooled together in an RNase-free falcon tube and considered as one biological replication. The pooled tissue sample was immediately frozen in liquid nitrogen and stored at -80°C until RNA extraction. Total RNA was extracted and purified using the Spectrum Plant Total RNA kit as recommended by the supplier (Sigma-Aldrich, Toronto, ON, Canada). The RNA pellet was diluted in 100 μl of RNase-free water supplemented with 1 μl of RNase OUT, and DNase-treated. An aliquot was visualized on an agarose gel before quantification using a Nanodrop (Thermo Scientific, Madison, WI, USA). The RNA sample was subsequently precipitated in 2 volumes of 100% cold ethanol supplemented with 0.1 volume of 3 M sodium acetate, pH 5.5, in 1.5 mL tubes and shipped on dry ice to Macrogen Inc (Macrogen Inc, South Korea) for library construction and sequencing. At reception, the RNA was re-pelleted and quality control was performed using a 2100 Bioanalyzer (Agilent Technologies). RNA QC was ensured as all samples had an RNA integrity number (RIN) ≥ 7.0 and absence of DNA contamination before moving for the library preparation and sequencing.

For the gene expression signaling and priming study from tubers collected in the greenhouse experiment, total RNA was extracted from 45 frozen tuber samples collected at the two time points as described earlier. The RNA quality was verified on agarose gel and an Experion (Bio-Rad, Mississauga, ON, Canada), and it was quantified using a Nanodrop (Thermo Fisher Scientific, CA, USA) before use in qPCR.

### Library construction and RNA sequencing

Paired-end RNA transcriptome sequencing was performed following Macrogen’s in-house workflow (Macrogen Inc, Seoul, South Korea). Briefly, One μg total RNA was used as starting material and cDNA libraries were generated using the Truseq stranded total RNA library preparation kit (Illumina, Inc, CA, USA). The libraries were quantified by qPCR and the paired-ends (2x100 bp) sequencing was performed on a HiSeq2500 platform (Illumina, Inc, CA, USA). The raw sequencing read data were submitted to the GenBank Short Read Archive (SRA) database and is available under the Bioproject ID# PRJNA532699.

### RNAseq data processing, clustering and differential gene expression

The integrity of the RNAseq raw read data was checked with FASQC V0.10.0 [37] and low quality (Phred Q scores < 20) and adapter sequences were trimmed using Trimmomatic V0.32 [38]. Reads with Phred quality score over 30 were assembled and mapped to the reference genome (GCF_00022675.1_SolTub_3.0) using Bowtie2 v2.2.3 [39] and Tophat v2.0.13 splice-aware aligner functions [40]. A maximum of five mismatches were allowed, insuring that each pair of forward and reverse reads mapped to the same transcript. The filtration parameters used were as described in [41]. Briefly, the default parameters were set at clustering precision = 0.95, span count threshold = 5, split count threshold = 3, percent identity threshold = 0.90, max dist pos = 600, num dist genes = 500, split min anchor = 4, max concordant ratio = 0.1, splice bias = 10, *de novo* assembly = no, probability threshold = 0.50. The GC content was determined, and the number of clean and mapped reads, as well as the mapping ratio, were scored. Transcriptome assembly, assignment, abundance and differential expression and regulation levels were inferred using Cufflinks version 2.2.1 [42] and the expression data were reported as fragment per kilobase of transcript per million mapped reads (FPKM) for each transcript/gene in each sample. The FPKM for each gene was calculated based on the length of the gene and reads count mapped to this gene. The G-option of Cufflinks, the reference annotation based transcript assembly (RABT) method of Cufflinks [43] and Cuffdiff [44] were used to determine the known gene and transcript expression levels, whereas the g-option of Cufflinks and the RABT assembly algorithm were used to investigate novel transcripts and novel alternatively spliced transcripts when they existed. Cuffdiff is part of Cufflinks and it calculates the expression in two or more samples and tests the statistical significance of observed expression change between samples [44]. The use of both Cufflinks and Cuffdiff was justified as they can automatically model and subtract a large fraction of the bias in RNAseq read distribution across transcript and improves abundance estimates [43]. The FPKM value was log2 transformed and quantile-normalized to produce more even data distribution and to reduce systematic bias from the pre-processed core libraries and reported as processed normalized FPKM. Differentially expressed genes (DEGs) analysis was performed based on the normalized processed FPKM data from the 6 paired-comparison including the three replicates of each of the two cultivars using scripts of the comprehensive R archive network (CRAN), version R 3.4.3 (http://cran.r-projetc.org). Statistical significance of the differential expression data was determined by performing independent t-test and fold change considering that there was no difference between groups as the null hypothesis. False discovery rate (FDR) was controlled by adjusting *P* value using Benjamini-Hochberg algorithm [45]. Genes that satisfied the absolute fold change (|fc|) of ≥2 and p<0.05 in the independent T-test from at least one of the paired-comparison were reported for each sample and transcript/gene. Correlation analysis, hierarchical clustering (Euclidian method, complete linkage), and multidimensional scaling analyses were performed using the heatmap.2 function provided by the R3.4.3 package gplots option for data visualization. Furthermore, functional classification, and gene-set enrichment per biological process, cellular component and molecular function analyses were performed using the **D**atabase for **A**nnotation, **V**isualization and **I**ntegrated **D**iscovery (**DAVID**) tool v6.8 [46,47] based on gene ontology (GO), Kyoto Encyclopedia of Genes and Genomes (KEGG, http://kegg.jp), and other functional annotation databases, including NCBI (http://ncbi.nlm.nih.gov), and uniprot (http://www.uniprot.org).

### RNAseq data validation by qPCR

To validate the RNAseq data, gene-specific primers were designed from 13 differentially expressed genes for qPCR (Table 1). The 13 genes are represented by 12 genes families. First strand cDNA was prepared from 600 ng total RNA for each of the three biological replicated tuber samples per cultivar and the qPCR reactions including 10 ng cDNA were performed on CFX96 Real Time system (BioRad, Laboratories, Canada) following the procedures described in a previous study [48]. Following the final amplification cycle, a melting dissociation curve was generated to ensure specificity of the primers and to confirm the uniqueness of the amplification product. The *18S rRNA* (X67238) gene was used as a housekeeping gene as previously suggested [49]. The output expression data were determined following the 2^-ΔΔCT^ method [50] and were reported as mean ±SD fold changes expression.

**Table 1:** List of primers used for RNAseq data validation using qPCR.

#	Protein_ID/ Ref Seq ID	Description	Primer sequence	Expected size (bp)	GC%
1	NP_001305476.1/	Cysteine protease inhibitor 8-like	TATGGTGATGTGGTGCGTCT	188	50
	NM_001318547.1		TTCATACCTCGTATCACCAAGTCA		42
2	XP_006353926.1/	Cysteine protease inhibitor 1	TGGTTGCCTTTGCTCGATCT	160	50
	XM_006353864.2		GCCCCGATGAGAGGATTGTT		55
3	NP_001275066.1/	L-ascorbate peroxidase 1, cytosolic-like	GCTCTCCTCTGTGATCCTGC	150	60
	NM_001288137.1		CAACTCCTCCTTCCCGTCAC		60
4	XP_006356407.1/	Endoglucanase 25-like	CAAGTTTGCCAGGGAACAGC	174	55
	XM_006356345.2		GCGTGTTTGGCAATACCAGG		55
5	XP_006349017.1/	Xyloglucan endotransglucosylase/hydrolase protein 31-L	ATCTTGGGCAACGGAGGAAG	194	55
	XM_006348955.2		GCACCCACAACATAGCCTCT		55
6	XP_015161134.1/	Chitin-binding lectin 1	ACGGCAATTAGCGTTTTAGCTC	196	45
	XM_015305648.1		TCCCGCCACCCTTTTTCAAT		50
7	XP_006367121.1/	Defensin J1-2	CACTCTTCTCCCTTCACCACA	172	52
	XM_006367059.2		CCCTTGAATCGACGGCTCTG		60
8	XP_006341994.1/	Protein WALLS ARE THIN 1	CCCTCAGGCTTGGCTAGTTC	179	60
	XM_006341932.2		CGAAGGAAGCCATCAGAGCA		55
9	XP_006356638.1/	BURP domain-containing protein 3-like	TCGAATACTTTTGTTGGCAGAGAA	150	40
	XM_006356576.2		TTCTCGGAGGCAGCCTGATA		55
10	XP_006366313.1/	7-deoxyloganetin glucosyltransferase-like	ATCCGAAACACCCCTAGTTGG	150	52
	XM_006366251.2		TGGTATTACATGACCTTGTGCAG		43
11	XP_015167139.1/	Putative late blight resistance protein homolog R1A-3	CCAGAATGAAACAATCAAGCTGC	200	43
	XM_015311653.1		TTGGCAGAGCAGAAAGCAGT		50
12	XP_006348827.1/	Transcription activator GLK1	CCACACCATCAACGGGTACA	190	55
	XM_006348765.2		ATTGTGCTGGAGGAGTAGCG		55
13	XP_015167354.1/	Cellulose synthase-like protein E1	CTGATTCCGAGTTACCGGGC	197	60
	XM_015311868.1		AGCAAATCCACATGCCTCCT		50

### Gene expression signaling and priming sensitization assessed by qPCR

To evaluate whether differential gene expression signaling and transgenerational priming sensitization [26] occur between GM and HB during the scab infection process, gene expression was monitored by qPCR using gene specific primers targeting six genes in tuber tissue collected in a controlled environment experiment (S1 Table). Total RNA from all the 45 tuber samples, representing two time-points and three biological replicates in the two cultivars, were used for the gene expression quantitation. The qPCR conditions and the housekeeping gene were the same as described for the RNAseq validation above, except that the amplification was performed at 61°C for all the genes. Gene expression data were plotted as normalized expression value to the housekeeping gene.

### Statistical analysis

Data from scab incidence and gene expression studies were analyzed using a mixed model analysis in GenStat (Release 12.1 for Windows). The random effects were units / time, with the fixed effects being cultivar * treatment * time. The differences between treatments were evaluated using an orthogonal contrast. Using the means from the mixed model analysis, a principal component analysis (PCA) was completed using correlations on Euclidian distances.

## Results

### Contrasting epiphytotic scab infection symptoms observed between Green Mountain and Hindenburg in the field conditions

After tuber harvest from the field and scab symptom rating, HB showed low percentage of scab incidence, surface coverage, low severity compared to GM (Fig 1; Table 2), confirming the well- known scab resistance status for HB and the susceptible status for GM [8,35]. No other visual disease symptoms were observed on the tuber surface of the two cultivars through the grading process. These observations provided confidence for conducting the transcriptomic studies.

**Table 2:** Common scab symptoms observed in Hindenburg and Green Mountain grown in heavily infested field plots at AAFC Harrington farm in 2016. The disease symptoms were rated and expressed as % incidence, % of tuber surface area covered and severity rating on a scale of 1 to 3.

Cultivar	Incidence (%)^a^	Surface covered (%)^b^	Severity^c^
Hindenburg	40	1.4± 0.0	1.0 ± 0.0
Green Mountain	100	67.5 ± 6.4	2.5 ± 0.0
Standard error	13.3	2.3	0

^a^Percentage of tubers with scab lesions,

^b^Percentage of tuber surface covered with lesions;

^c^Severity scale: 1 = superficial lesions; 2 = raised and corky lesions; 3 = pitted lesions. Number represents means ± standard deviation from four replicates.

### Field epiphytotic scab symptoms in Green Mountain and Hindenburg confirmed by the greenhouse experiment

**Fig 3:**
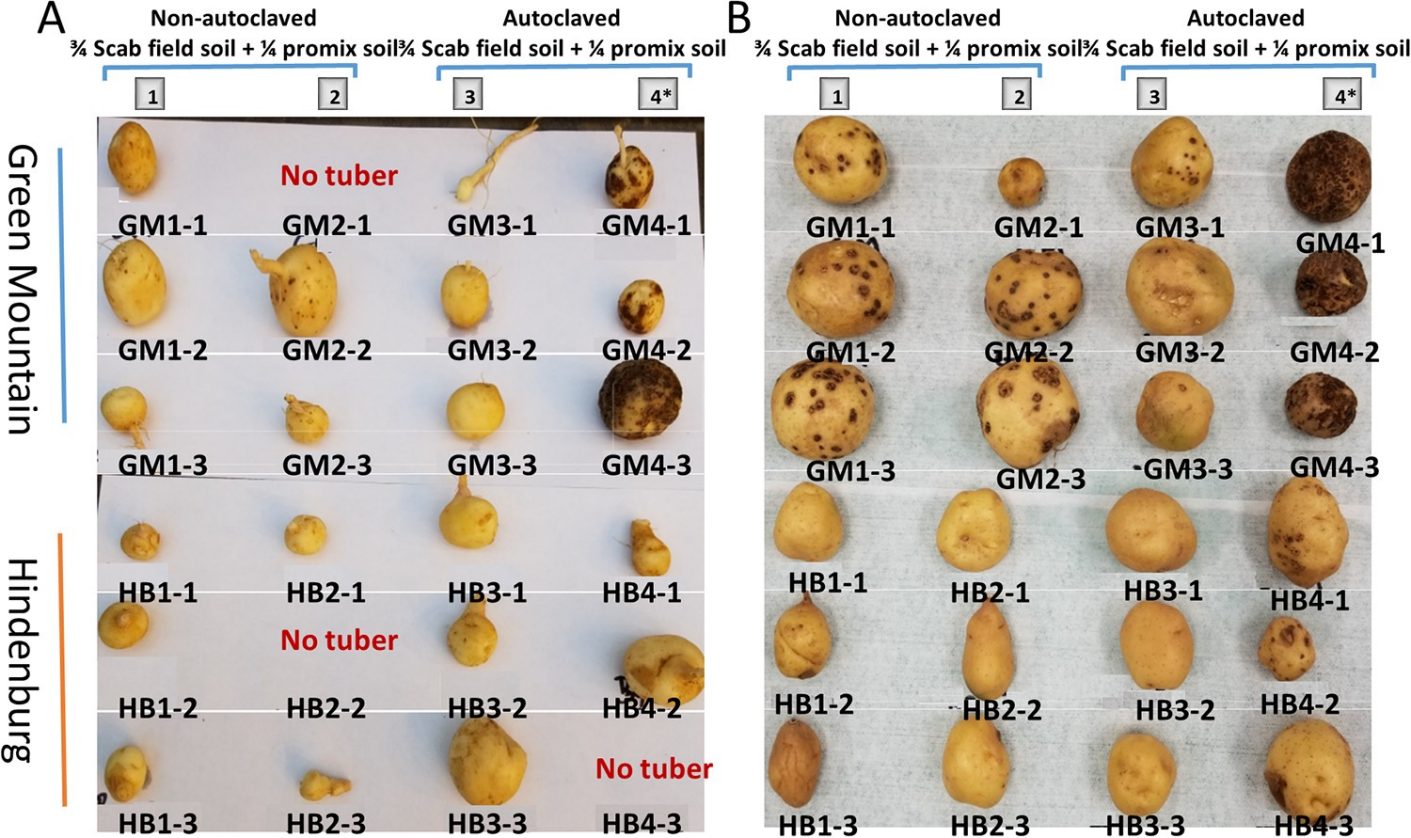
Scab symptoms (dark dots) as observed in Green Mountain (GM) and Hindenburg (HB) grown in a greenhouse experiment under four treatments consisting of a combination of three soil and two seed types. **A)** Scab symptoms on tubers collected from GM and HB at time point 1; **B)** Scab symptoms on tubers collected from GM and HB at time point 2.

To ascertain whether the observed scab symptoms in the field were only due to natural scab populations but not other pathogens, plants were grown in a greenhouse in winter 2019. After harvesting and grading, scab symptoms were observed only in treatments 1 (naClean), 2 (naScab) and 4 (acInoScab) at both time points 1 and 2 (Fig 3A and 3B). At time point 1, small brown scab symptoms were observed at the tuber surface of cultivar GM but not for HB when grown in the non-sterile soil (Treatment 1 and 2). These symptoms were more obvious and larger on tubers collected from the sterile soil inoculated with the scab pathogen (Treatment 4). No obvious lesions could be seen in HB at time point 1 (Fig 3A). At time point 2 in contrast, scab symptoms were more obvious, and clear differences could be observed between cultivars GM and HB, as well as between treatments with infected soils (Treatments 1, 2, 4) and the sterile soil treatment 3. In this later treatment (Treatment 3), only very few small scab lesions could be observed in a single replicate of GM, but none of HB (Fig 3B). In treatment 4, whereas tubers from GM were fully covered with scab symptoms, only very little scab reactions were seen in HB (Fig 3B). Thus, a three-way interaction was observed between treatments by (GM and HB) and both time points (P<0.001). A significance difference (P<0.001) was also observed between treatments, cultivars and time points for all disease rating criteria (Table 3).

**Table 3:** Scab rating from four treatments, two time points and two potato cultivars GM (susceptible) and HB (resistant) in a greenhouse experiment. ANOVA test levels are shown. Rating parameters were variates and treatment, cultivar and time point were taken as fixed effects.

Disease rating	Treatment*						Cultivar				Time point			
	naClean	naScab	acClean	acInoScab	Mean	P<F	GM	HB	Mean	P<F	Time 1	Time 2	Mean	P<F
Incidence	30.7	40	25	58	38.4	***	66.2	10.7	38.4	***	16.8	60	38.4	***
Coverage	10.3	14.2	5.4	39.9	17.5	***	34.4	0.6	17.5	***	8.5	26.5	17.5	***
Severity	0.9	1.1	0.6	1.7	1.1	***	1.8	0.3	1.01	***	0.5	1.7	1.1	***

*The four treatments named as naClean (non-autoclaved soil planted with clean seed), naScab (non-autoclaved soil planted with scab infected seed), acClean (autoclaved soil planted with clean seed), acInoScab (autoclaved soil inoculated with scab pathogen and planted with scab infected seed) correspond to treatments 1, 2, 3 and 4 as in Fig 1, respectively.

***indicates significance levels at P<0.001.

### RNAseq mapping data statistics

Whole RNA transcriptome libraries were successfully built from three biological replicated tuber samples collected in the field from the scab-resistant potato cultivar HB and the susceptible cultivar GM. High and consistent quality read numbers were observed in each of the triplicated samples of the two cultivars investigated. On average, a total of 35.4 million pair-end raw reads, 35.0 million cleaned pair-end reads, and 14.8 million mapped mate-pair reads to the reference genome per sample were generated (Table 4). The mapping ratio ranged from 80 to 87% with an average of 84%. The proportion of multiple mapped reads ranged from 8.7% to 23.1%, with the lowest percentage observed in the library generated from replicate 1 of HB and the highest in the replicate 3 of GM. The proportion of multiple mapped reads in the remaining replicates of the two samples ranged from 15.2% to 21.5% (Table 4).

**Table 4:** Summary statistics of the RNAseq transcriptome sequencing reads and mapping to the reference genome.

		Raw read data statistics^a^			Trimmed read data statistics^a^				Mapped read data statistics^b^			
Cv	Rep	Total bases	Total reads	Q30 (%)	Total bases	Total reads	GC (%)	Q30(%)	Processed Reads^c^	Mapped Reads^d^	Multiple mapped reads^e^	Overall read mapped (%)^f^
GM	1	3,492,844,216	34,582,616	96	3,462,970,693	34,359,880	47	96	17,179,940	14,752,617	2,393,750	85
	2	3,628,544,382	35,926,182	96	3,602,196,724	35,744,342	46	96	17,872,171	14,823,012	2,530,422	82
	3	3,631,650,536	35,956,936	95	3,486,424,735	34,626,526	48	96	17,313,263	15,155,651	3,494,252	86
HB	1	3,719,750,816	36,829,216	96	3,697,410,480	36,684,900	44	96	18,342,450	14,806,752	1,281,447	80
	2	3,478,622,406	34,441,806	96	3,449,773,807	34,237,688	47	96	17,118,844	14,653,925	3,156,878	85
	3	3,484,634,936	34,501,336	96	3,456,979,908	34,302,374	46	96	17,151,187	14,465,389	2,204,594	84
**Av**		**3,572,674,549**	**35,373,015**	**96**	**3,525,959,391**	**34,992,618**	**46**	**96**	**17,496,309**	**14,776,224**	**2,510,224**	**84**

^a^Statistics on total pair-end reads;

^b^Statistics on total mate-pair reads;

^c^Number of cleaned reads after trimming;

^d^Number of reads mapped to reference;

^e^Number of reads multiple mapped to reference;

^f^Number of total mapped reads/number of total processed reads.

### Differential gene expression

**Fig 4:**
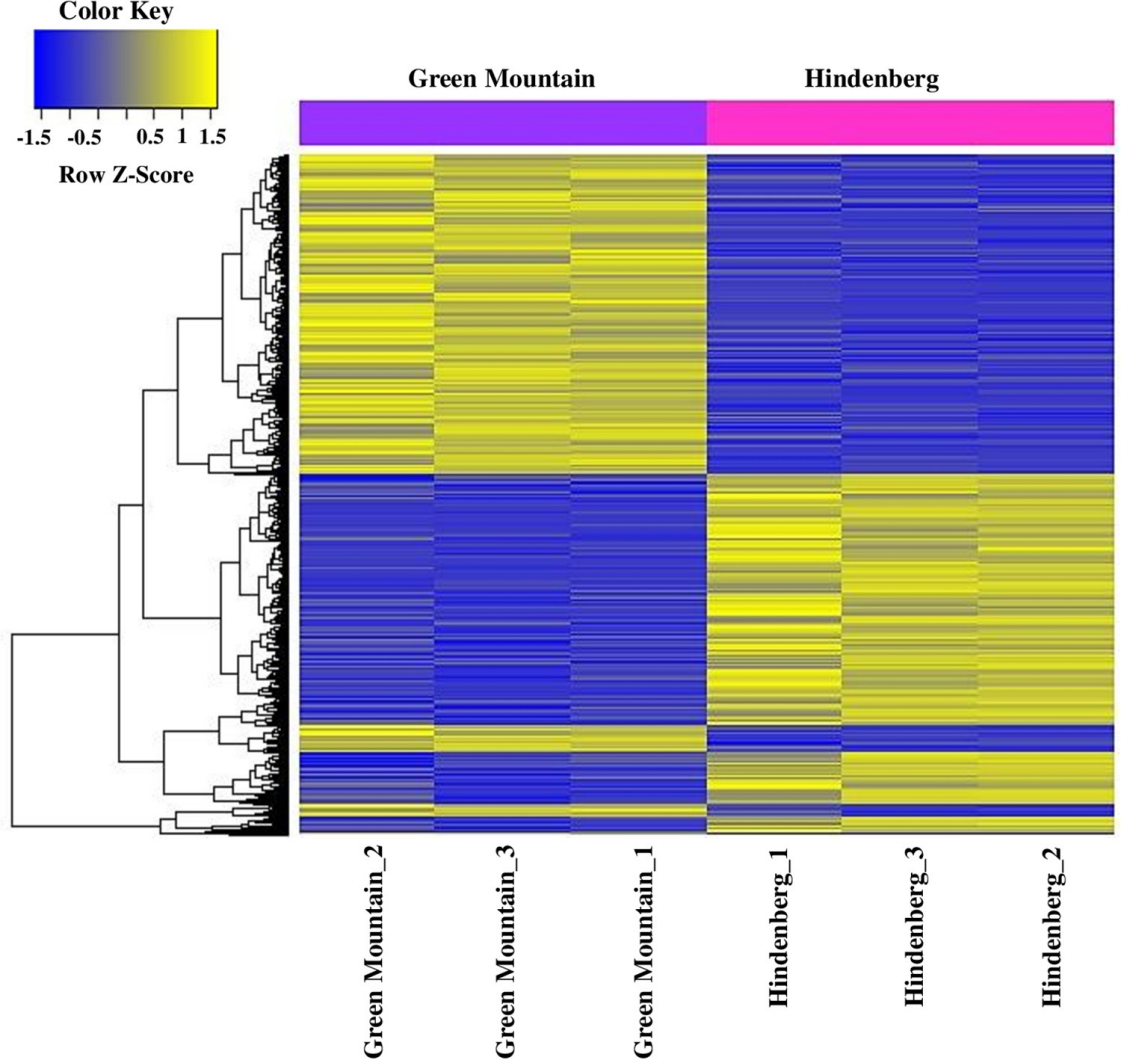
Hierarchical clustering heat map showing differentially expressed genes in triplicated samples of the scab susceptible cultivar Green Mountain and the resistant cultivar Hindenburg. Complete linkage and Euclidian distance were used as a measure of similarity to display expression patterns of DEGs with absolute fold change ≥2. Each row corresponds to a gene and each column corresponds to an individual replicate for each cultivar. The cultivar GM and HB are color-coded in purple and magenta, respectively. The normalized expression z-score scale is indicated by the color key in the top left corner. Blue, down-regulated genes; Yellow, up-regulated genes.

To assess the transcriptomic expression responses of the resistant HB and susceptible potato cultivar GM to common scab in the mature tubers, a comparative differential gene expression analysis was performed through RNA sequencing. After read mapping, transcript assembly, data transformation and quantile normalization, a total of processed 25,548 annotated expressed genes were observed in at least one replicate of the two cultivars (S2 Table). Among these genes, *MPK3* (XP_006352951.1, XP_006353717.1) and serine threonine kinase *EDR1* (XP_006348875.1, XP_006365408.1, XP_006341569.1, XP_006362406.1), salicylic acid binding protein (XP_015160896.1, XP_015163790.1, XP_006346477.1,XP_006346479.1, XP_006346475.1) were found to be 2–7X more abundantly expressed in HB than in GM (S2 and S3 Tables). More expressed known novel transcripts (11,044 *vs* 9,399) and novel spliced variants (5,703 *vs* 4801) were also observed in HB than in GM, accounting on average for 17% more known novel transcripts and 18% more novel spliced variants in HB. Among the four novel spliced isoforms of thioredoxin 1 like protein observed in the study, three (XP_006348246.1, XP_006358980.1, XP_006366027.1) showed more transcripts in HB than in GM, and the fourth (XP_006362954.1) was down-regulated in HB. Moreover, the chloroplastic thioredoxin like 1–1 was represented by three isoforms, two of which (XP_006354773.1 and XP_006351368.1/XP_006351369.1) were highly expressed in HB compared to GM, whereas the third isoform (XP_006348023.1) had the same expression level in the two cultivars. Differential expressions of *Mlo* and *Miraculin like* (*Mpl*) gene transcripts were also observed (S2 and S3 Tables) and the data revealed 159 novel transcripts in HB whereas 115 were found in GM. Analyzing the spliced transcript variants, 3,361 and 2,251 spliced variants were found in HB and GM, respectively, with 1,073 spliced variants shared between the two cultivars. These spliced variants were found to code for 3,181 and 1,881 proteins in HB and GM, respectively, with 1,404 proteins shared between the two cultivars (S1A and S1B Fig). After further statistical analyses of the 25,548 annotated gene dataset, a total of 1,064 differentially expressed genes showing an absolute fold change value of ≥2 and P<0.05, were observed across all the 6 replicates in the two cultivars, and a hierarchical clustering heat map depicted a clear contrasting DEG pattern between the potato cultivars (Fig 4).

**Fig 5:**
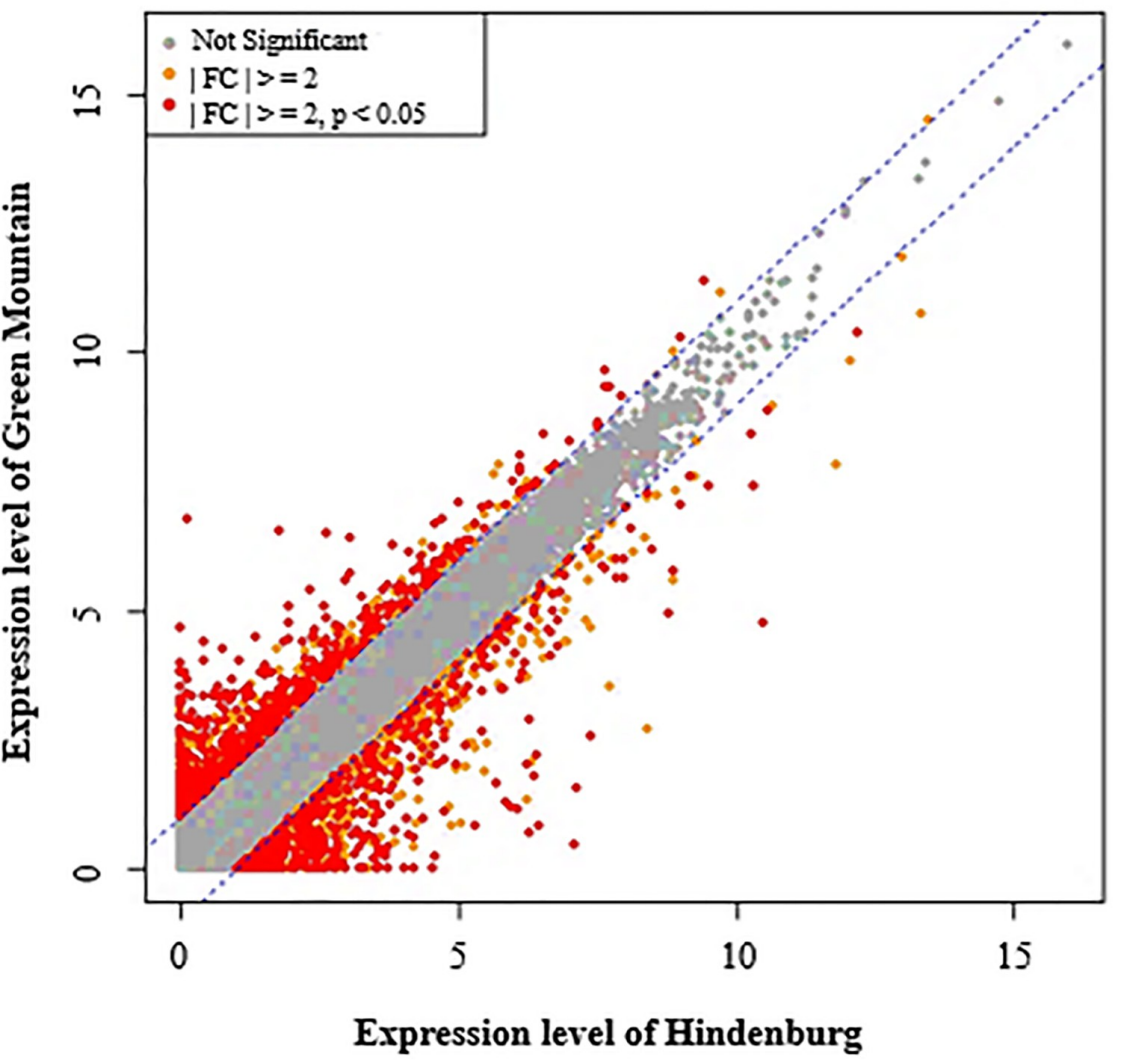
Scatter plot of gene expression levels in the scab susceptible cultivar Green Mountain and resistant cultivar Hindenburg. Genes with absolute fold change ≥2 and P<0.05 are indicated by red dots. Genes with absolute fold change ≥2 but not significant at P<0.05 level are indicated by yellow dots, and genes with unchanged gene expression in the two cultivars are indicated by grey dots.

**Fig 6:**
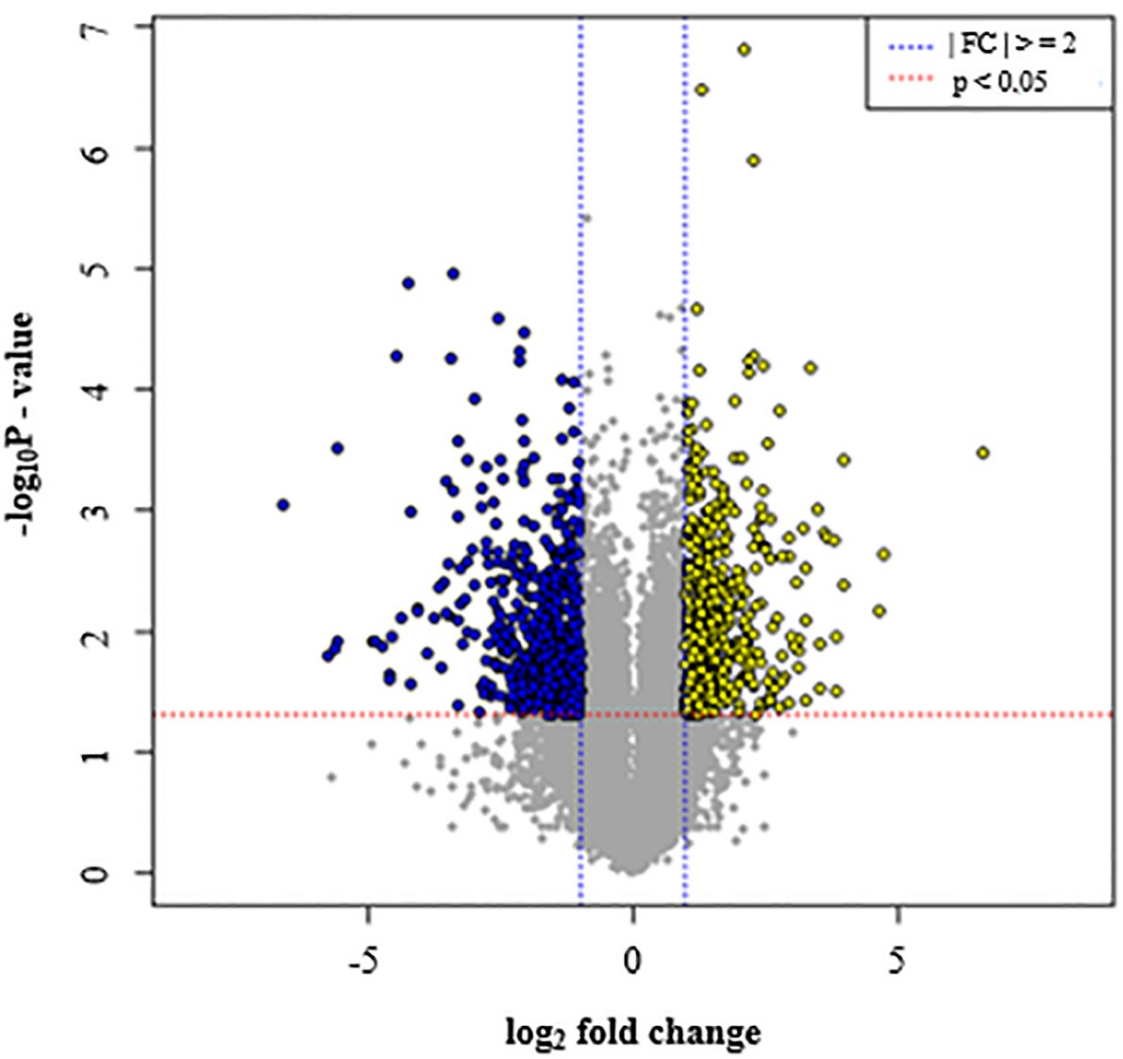
Volcano plot of gene expression levels in the scab susceptible cultivar Green Mountain and resistant cultivar Hindenburg. Log2 change and P-value obtained from the comparison of the average for each group were plotted as volcano plot. Significant genes which absolute fold change ≥2 and P<0.05 are indicated by blue and yellow dots.

From the DEGs across the six replicates, a total of 501 genes were found to be down-regulated 2.0 to 99.0 folds in GM compared to HB, whereas 563 genes were up-regulated by 2.0 to 99.0 folds in GM compared to HB (S3 Table). These DEGs included putative disease resistance genes (XP_015164732.1), *RPP13* (XP_015169822.1) and *RGA4* (XP_015164580.1, XP_006338949.1), LRR receptor-like kinase (XP_015159898.1, XP_006356742.1, XP_006358074.2, XP_006348920.1, XP_015169250.1, XP_006354051.1) involved in plant immune defense responses, salicylic acid binding proteins (XP_015160896.1, XP_006346475.1), as well as transcription factors such as MYB, WRKY and BHLH (XP_006357746.1). Indeed, two differentially expressed LRR receptor-like serine/threonine-protein kinase isoforms (EFR and FEI 2) were observed, with isoform EFR (XP_015160746.1) being 2-folds up-regulated in GM whereas isoform FEI 2 (XP_015159898.1) was 2-folds down-regulated in GM. In addition, the expression of Subtilisin-like protease SBT1-7 (XP_006365833) was found to be 7X downregulated in GM compared to HB (S3 Table). Furthermore, a relatively small proportion of genes showing fold change ≥ 2, but not significant (P>0.05), was also observed in the 2 cultivars along with genes of unchanged levels of expression (Fig 5). The DEG data were further subjected to volcano plot data analysis for estimating the size effect and significance of each tested gene and a clear representation of down and up-expressed genes in GM versus HB was highlighted (Fig 6).

### Functional classification and gene enrichment

**Fig 7:**
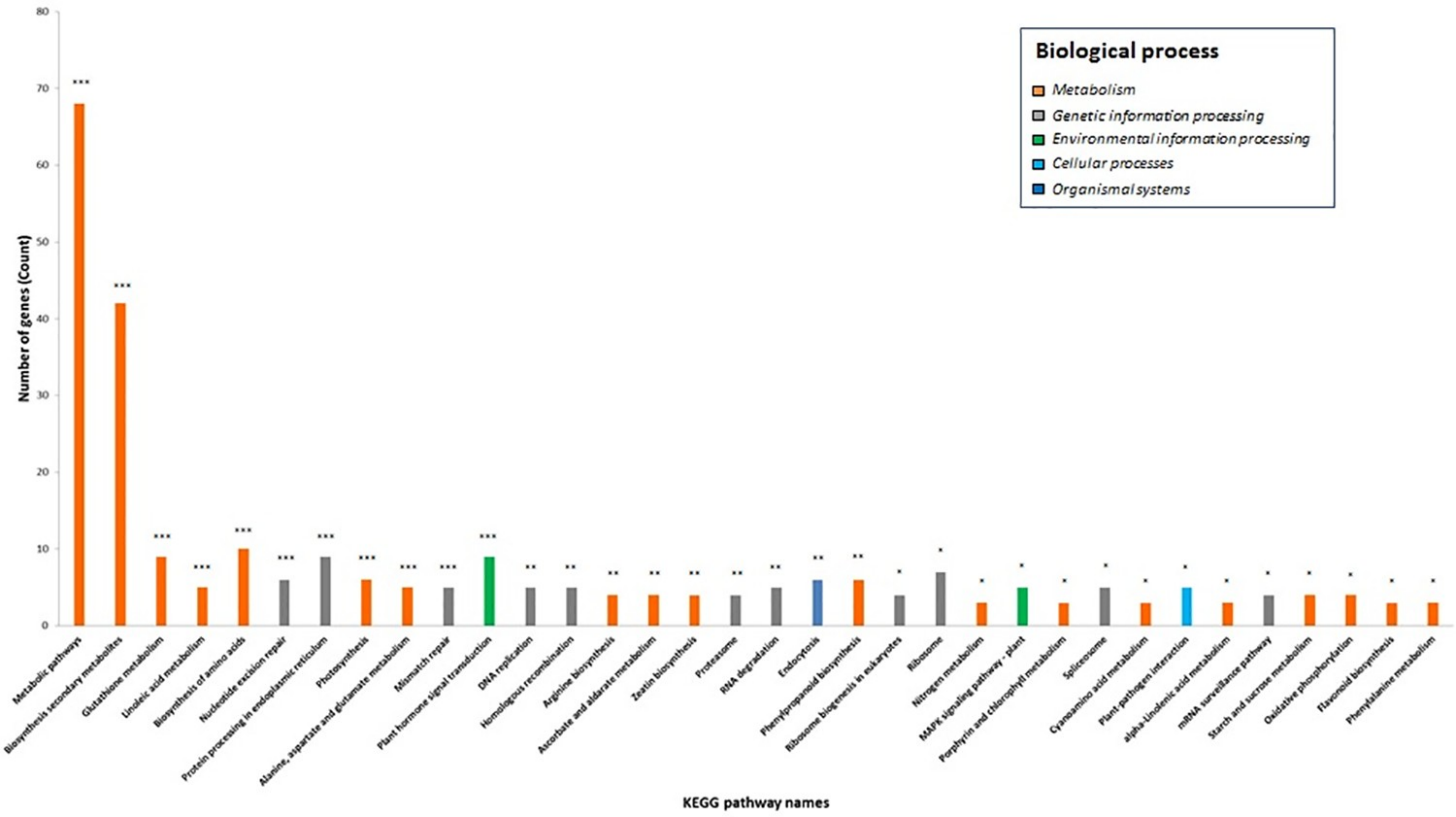
KEGG pathways assignment to DEGs in the scab susceptible cultivar Green Mountain and resistant cultivar Hindenburg. A total of 273 enriched DEGs were assigned to 34 KEGG pathways with high confidence. Levels of significance are indicated by red asterisks with *, **, and *** for P<0.05, P<0.01, and P<0.001, respectively.

To identify GO categories, classify and assign biological functions associated with the DEGs, GO and KEGG pathway enrichment analysis were performed using DAVID tools. Of the 1,064 differentially expressed genes across all six replicates, 273 were assigned with high confidence (P<0.05 and FDR<0.05) to 34 KEGG pathways, and genes involved in metabolic pathways and biosynthesis of secondary metabolites accounted for 68% of the 273 DEGs (Fig 7).

**Fig 8:**
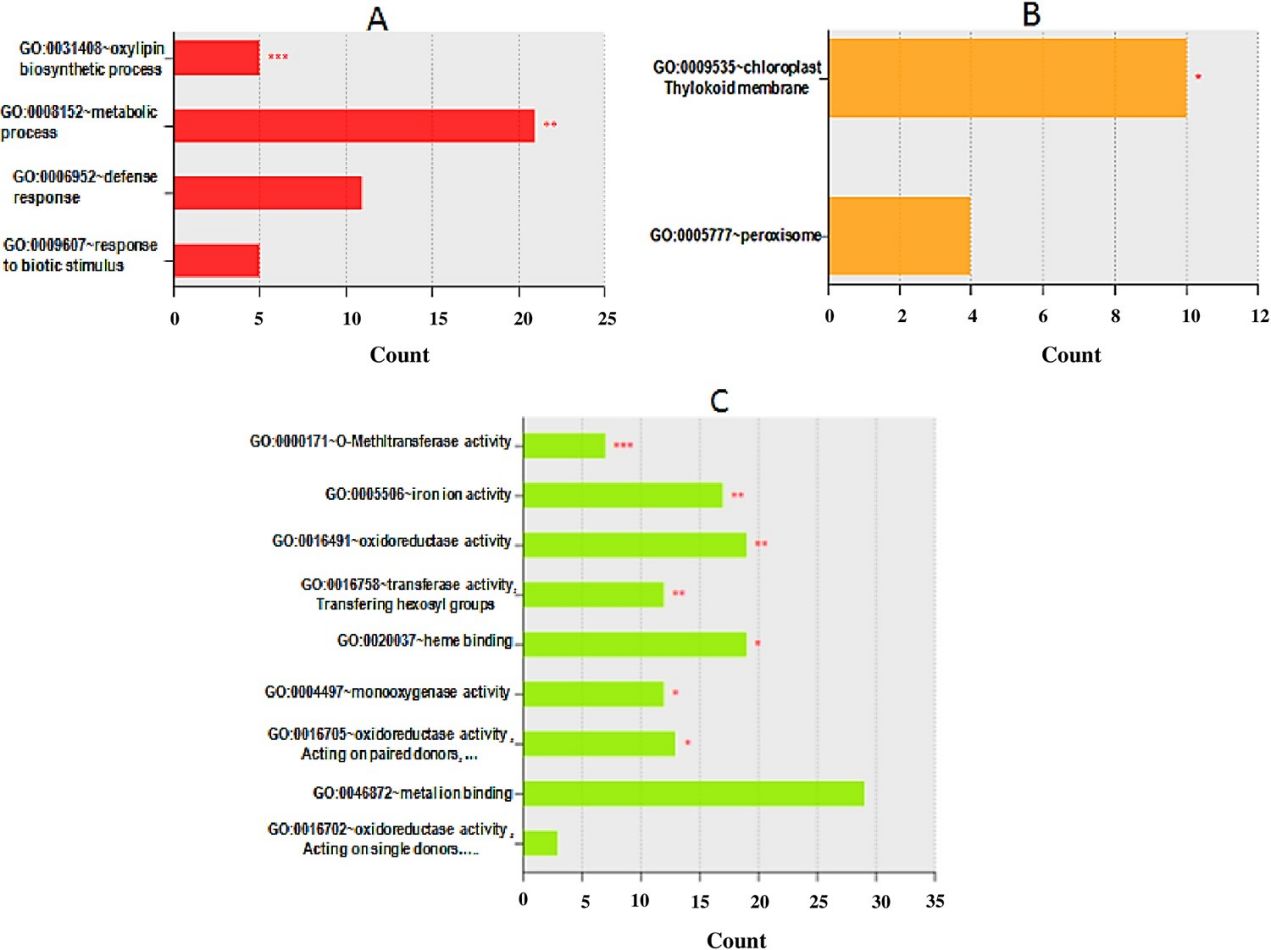
Bar plot representation of the GO term gene enrichment of DEGs between the scab susceptible cultivar Green Mountain and resistant cultivar Hindenburg using David tool. **A**, biological process; **B**, Cellular component; **C**, Molecular function. Levels of significance are indicated by red asterisks with *, **, and *** for P<0.05, P<0.01, and P<0.001, respectively.

By further analyzing the DEGs using David tools, a total of 4, 2, and 9 GO terms were found to be associated with biological processes, cellular component, and molecular functions, respectively (Fig 8). The biological processes related to environmental information processing that includes plant-pathogen interaction and response to biotic stimulus was found to be differentially expressed between GM and HB (Figs 7 and 8). A detailed pathway analysis showed that genes involved in N-glycan, steroid, terpenoid, sesquiterpenoid and triterpenoid, phenylpropanoid, flavonoid, tropinone, piperidine, and pyridine biosynthesis, as well as those involved in pathogen-sensing and priming such as MAPK kinase signaling and plant-pathogen interactions, were found in majority to be down-regulated in GM compared to HB (Table 5). Of particular interest was the coordinated down-regulation of genes in the brassinosteroid biosynthesis within the terpenoid pathway (S2 Fig).

**Table 5:** Summary table of genes in key pathways found to be altered in GM compared with HB.

KEGG ID#	Pathway names	Gene names	Regulation status	
			GM	HB
Sot00073	Cutin, suberin and wax biosynthesis	cytochrome P450 94A2-like/ fatty acid omega-hydroxylase	up	Down
Sot00100	Steroid biosynthesis	squalene synthase-like; delta(24)-sterol reductase-like; putative C-8,7 sterol isomerase; 7-dehydrocholesterol reductase-like; cycloeucalenol cycloisomerase; delta(14)-sterol reductase; putative C-8,7 sterol isomerase	down	Up
Sot00130	Ubiquinone /terpenoid-quinone biosynthesis	gamma-tocopherol methyltransferase	up	Down
Sot00190	Oxidative phosphorylation	NAD(P)H-quinone oxidoreductase subunit 6	down	Up
Sot00510	N-Glycan biosynthesis	alpha-1,3-mannosyl-glycoprotein 2-beta-N-acetylglucosaminyltransferase	down	Up
Sot00563	Glycosylphosphatidylinositol (GPI) anchor biosynthesis	phosphatidylinositol N-acetylglucosaminyltransferase subunit P-like	down	up
Sot00591	Alpha linoleic acid metabolism	linoleate 9S-lipoxygenase	down	Up
Sot00592	Alpha linolenic acid metabolism	fatty acid hydroperoxide lyase	up	Down
Sot00592	Alpha linolenic acid metabolism	allene oxide cyclase	down	Up
		jasmonate O-methyltransferase		
Sot00600	Sphingolipid metabolism	neutral ceramidase-like	down	Up
Sot00730	Thiamine metabolism	ribosome biogenesis GTPase/thiamine phosphate phosphatase	down	Up
Sot00900	Terpenoid backbone biosynthesis	2-C-methyl-D-erythritol 2,4-cyclodiphosphate synthase, chloroplastic	down	Up
Sot00908	Zeatin biosynthesis	adenylate isopentenyl transferase 3, chloroplastic-like	up	Down
Sot00909	Sesquiterpenoid and triterpenoid biosynthesis	farnesyl-diphosphate farnesyltransferase	down	Up
Sot00940	Phenylpropanoid biosynthesis	caffeoylshikimate esterase	down	Up
Sot00941	Flavonoid biosynthesis	flavonoid 3'-monooxygenase	down	Up
		cinnamoyl CoA reductase		
		probable caffeoyl-CoA O-methyltransferase		
		coniferyl-alcohol glucosyltransferase		
Sot00960	Tropane, piperidine and pyridine alkaloid biosynthesis	Putrescine N-methyltransferase/tropinose reductase	Down	Up
Sot00970	Aminoacyl-tRNA biosynthesis	tRNA-amino acyl	down	Up
Sot03008	Ribosome biogenesis in eukaryotes	DKC1, Rrp7, LSG1	up	Down
Sot03015	mRNA surveillance pathway	cleavage and polyadenylation specificity factor ICPSF3 and CPSF5	up	Down
Sot03020	RNA polymerase	DNA-directed RNA polymerase III subunit rpc6	up	Down
Sot03022	Basal transcription factors (Eukaryotes)	general transcription factor IIH subunit 2-like	up	Down
Sot03430	Mismatch repair	DNA mismatch repair protein MLH3	down	Up
Sot04016	MAPK signaling pathway	MAPK8	down	Up
Sot04122	Sulfur relay system	cytoplasmic tRNA 2-thiolation protein 1 (NCS6)	up	Down
Sot04146	Peroxisome	peroxisomal coenzyme A diphosphatase NUDT7	down	Up
Sot04626	Plant pathogen interaction	pathogenesis-related genes transcriptional activator PTI4	down	Up
		pto-interacting protein 1		
		pathogen-induced protein kinase		

### RNAseq data validation by qPCR

**Fig 9:**
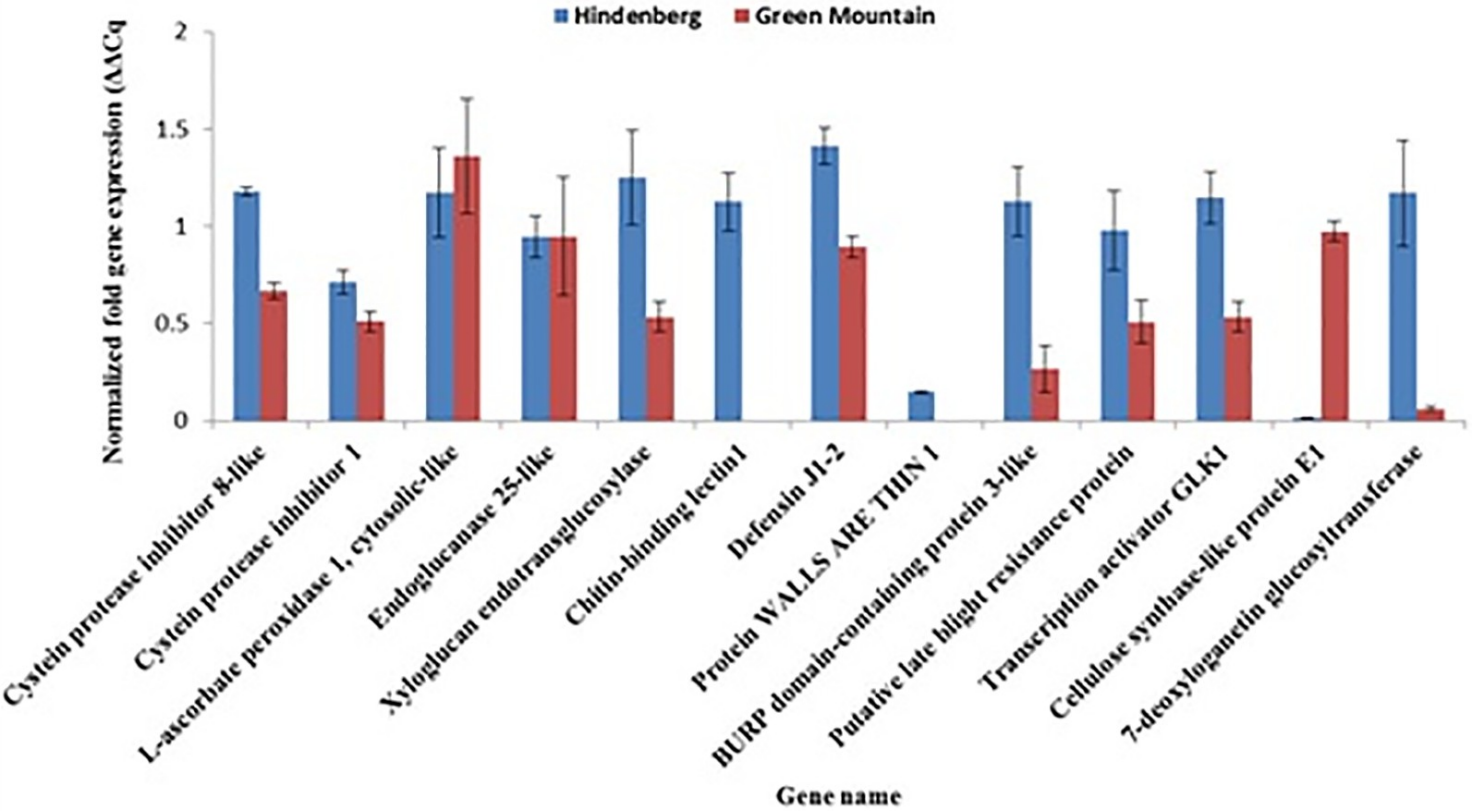
qPCR expression profiles for thirteen selected genes from the RNAseq dataset for validation. The expression data are expressed as normalized fold change to the 18S RNA expression and shown as the mean of three independent replicates. Vertical bars represent the standard deviation of the means.

The RNAseq data validation was performed by qPCR using 13 genes representing 12 classes of genes. qPCR expression patterns from 11 of the tested genes were consistent with the RNAseq profiling (Table 6, Fig 9). The expression levels of ascorbate peroxidase and endoglucanase were not significantly different in the two cultivars. Except for cellulose synthase-like protein E1 showing a down-regulation in HB as was the case in the RNAseq dataset, the remaining 10 genes were all found by the qPCR to be up-regulated in HB and down-regulated in GM as observed in the RNAseq profiling. Under the experimental conditions described, no transcript was detected in GM for *Chitin binding lectin 1* and in the *Protein walls are thin 1* gene in contrast to HB (Fig 9).

**Table 6:** RNAseq gene expression validation using by qPCR. Fold change expression observed by qPCR is shown along with the observed expression in RNAseq profiling.

#	Protein_ID	Description	Fold change (GreenMount_S/Hindenburg_R by RNAseq	Ratio GreenMount_S/Hindenburg_R by qPCR	Fold change (GreenMount_S/Hindenburg_R) by qPCR
1	NP_001305476.1	Cysteine protease inhibitor 8-like	-3.48	0.68	-1.47
2	XP_006353926.1	Cysteine protease inhibitor 1	-3.32	0.47	-2.13
3	NP_001275066.1	L-ascorbate peroxidase 1,cytosolic-	-2.44	1.16	1.16
4	XP_006356407.1	Endoglucanase 25-like	-5.05	1.00	1.00
5	XP_006349017.1	Xyloglucan endotransglucosylase/hydrolase protein 31-like	-5.46	0.42	-2.38
6	XP_015161134.1	Chitin-binding lectin 1	-49.93	0.01	-100.00
7	XP_006367121.1	Defensin J1-2	-4.86	0.63	-1.59
8	XP_006341994.1	Protein WALLS ARE THIN 1	-6.28	0.01	-100.00
9	XP_006356638.1	BURP domain-containing protein 3	-30.09	0.24	-4.17
10	XP_006366313.1	7-deoxyloganetin glucosyltransferase-like	-47.64	0.05	-20.00
11	XP_015167139.1	Putative late blight resistance protein homolog R1A-3	-3.63	0.52	-1.92
12	XP_006348827.1	Transcription activator GLK1	-4.84	0.47	-2.13
13	XP_015167354.1	Cellulose synthase-like protein E1	**13.91**	**25.10**	**25.10**

### Hindenburg and Green Mountain have different gene signaling and priming sensitization reactions to scab

Gene expression profiling performed using six target genes showed a time-dependent gene induction in HB for *MPK3*, *EDR1*, *Subtilisin*, and *MLO13* in all treatments, but no such clear relationship was observed for *MLO1* and *MLO8*, particularly in treatment 3 (S3 Fig). In contrast, no consistent time-dependent trend in differential gene expression and signaling was observed between treatments for GM at none of the time points, with the exception for *Subtilisin*, for which a time-dependent induction was observed. At time point 1, expressions of *MPK3*, *EDR1*, *Subtilisin*, *MLO1*, and *MLO8* were higher in GM compared to HB for most treatments excluding treatment 3. The expression of these genes consistently increased in HB at time point 2 (S3 Fig). Statistical analyses showed significant difference between cultivars for *EDR1* (P<0.001), *MLO1* (P<0.01) and *MLO8* (P<0.05) expressions, but not for *MLO13*, *MPK3*, and *Subtilisin*. Treatment effects on gene expression was significant for *MLO13* and *MLO8* (P<0.05), and for *MPK3* (P<0.01). However, while differences were observed for *MLO1* and *Subtilisin* expressions, the difference was not significant (P = 0.08). The interaction between treatment and cultivar was significant (P<0.05) only for *MLO1*. A time-dependent effect was observed only for *EDR1* (P<0.05), *MPK3* (P<0.001) and *Subtilisin* (P<0.001) gene expression levels.

**Fig 10:**
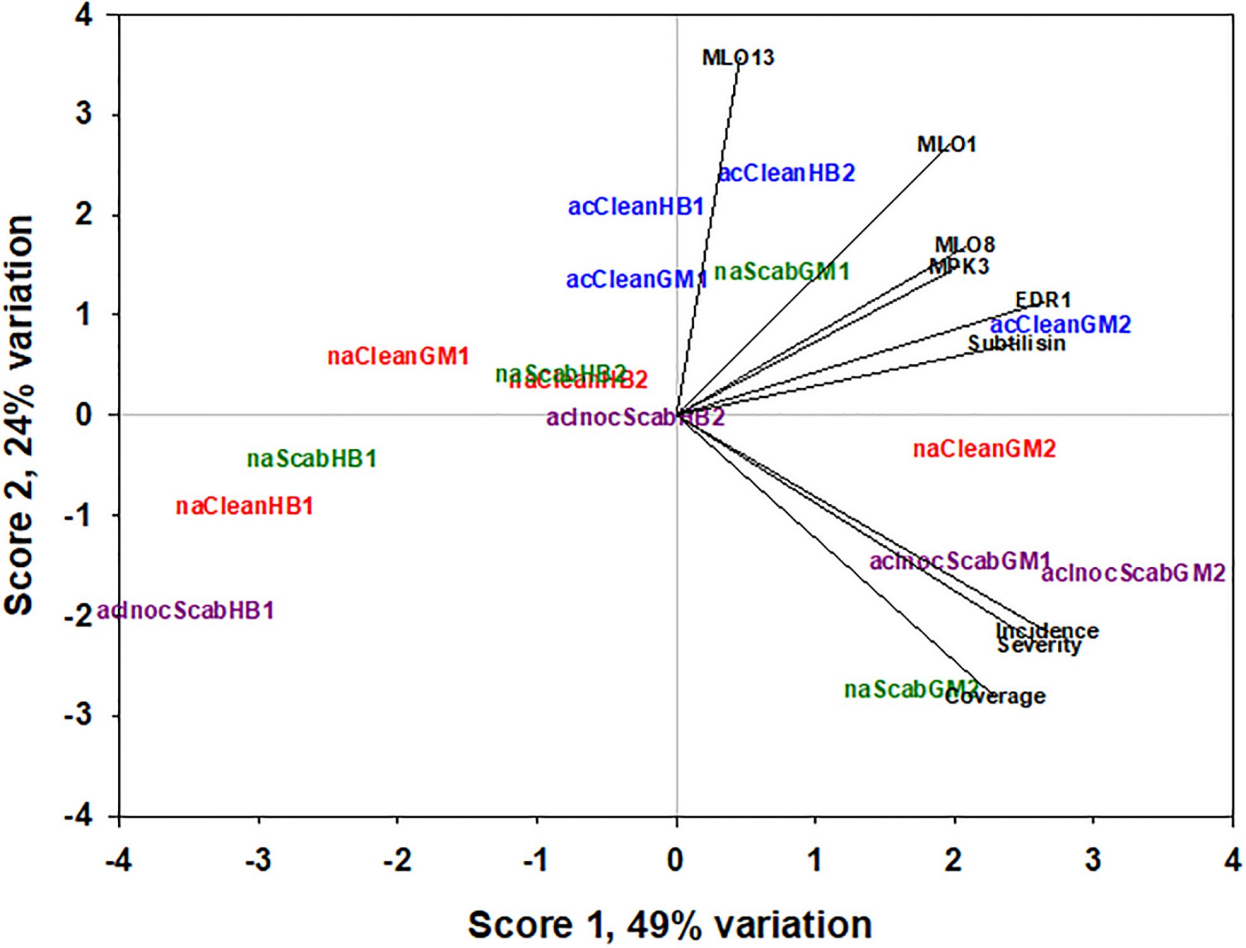
Principal component analysis (PCA) plot showing association between time-course of signaling and priming-associated genes gene expressions at time point 1 and 2 with scab disease symptom rating. Only variations explained by PCA score 1 and 2 are shown.

To see whether there was an association between treatments, gene expression profiles and scab disease rating, a principal component analysis (PCA) was performed. The results showed that gene expressions of *EDR1* and *Subtilisin* were the most correlated to the disease symptoms, and were further found to be closely associated with the treatments involving GM, the susceptible line (Fig 10). Using the PCA scores 1 and 2, explaining 49% and 24% of the variations, respectively, it was worth noting that the three treatments (naScabHB1, naCleanHB1, and acInoScabHB1) that scored null to very low scab symptoms and expressed low *EDR1*, *MPK3* and *Subtilisin* gene transcripts at time point 1 were grouped as separate. The low to moderately infected treatments (naCleanGM1, naScabHB2, naCleanHB2, acInoScabHB2, acCleanHB1, acCleanHB2, acCleanGM1, naScabGM1) were also grouped together in the center of the PCA plot whilst the most severely infected treatments involving GM were clustered with the diseases symptoms (Fig 10). Similarly, the PCA scores 1 and 3 (explaining 11% of the variations) also indicate that *EDR1* and *Subsitilin* were the most closely related with the disease symptoms and that *MPK3* and *MLO8* seem to be opposite each other (S4 Fig).

## Discussion

Common scab management and control in potato production areas have become a top priority for producers [1]. Soil pH management and crop rotation can reduce scab density, but they do not suppress the disease [2,9]. A viable and sustainable scab management strategy targets the development of scab-resistant potato cultivars [19] as well as the identification of common scab’s antagonists as biocontrol agents [1,14,51]. In the current study, a comparative RNA transcriptome sequencing validated by qPCR and induced gene signaling studies were conducted using two potato cultivars different by their response to scab for the purpose of unraveling genes and pathways differentially activated in this pathosystem. This study showed a consistent and contrasted DEG pattern between the two cultivars, identified a set of 273 differentially expressed genes from 34 enriched KEGG pathways, and demonstrates that HB has a higher pathogen-induced immune sensitization capacity than GM. It suggests a transcriptional immune priming activation in HB for this pathosystem, likely using salicylic-dependent signaling pathway, as a form of a memory-based mechanism [28] for induced-resistance in the scab resistant potato cultivar HB.

### Contrasting DEGs between HB and GM reflects the different genetics and disease reactions

By using RNA transcriptomic analysis of two potato cultivars grown under field conditions, shared transcript information across gene sets from three biological replications was produced to ensure direct comparisons. Accurate and precise transcript variance estimates as well as multiple pairwise comparisons were inferred for generating a robust dataset [52]. Our data showed clear and consistent DEG patterns that distinguished HB from GM. The observed differential transcript patterns appeared to reflect the genetic make-up of the cultivars and the phenotypic variations of disease symptoms observed at the tuber’s surface. A set of 273 genes from 34 enriched KEGG pathways, including metabolism and biosynthesis of secondary metabolites, as well as in biological processes and molecular function related environmental information processing, were mostly found to be down-regulated in GM, but up-regulated in HB. The data suggest that comparative transcriptomic phenotyping can be used to predict scab lesion phenotype in breeding lines. By speculation, it is indeed expected that lines displaying similar transcript patterns will show similar disease reactions. In fact, if the inherited genetic armories contributing to the resistance phenotype are present in a genotype, these defensive systems should be in a constant alert and be tuned to respond at any developmental stage to aggressions through an intrinsic induced-immune resistance [30,53] and thus their transcriptional patterns should be different from genotypes lacking these genetic features as it seems to be the case in the current study for HB and GM. A full genome sequencing of the two cultivars will however be needed to get more insights.

### Altered secondary metabolism gene expression between HB and GM

In the current study, worth noting was the differential expression of genes involved in steroid, N-glycan, terpenoid, sesquiterpenoid and triterpenoid, phenylpropanoid, flavonoid, tropinone, piperidine and pyridine biosynthesis as well as genes involved in plant-pathogen interaction and perception of biotic stimuli signaling. Differential transcriptional changes between the resistant and susceptible cultivars for genes in the phenylpropanoid pathway support the idea that suberin’s phenolic composition and the level of suberization play essential roles in scab resistance [17]. In fact, phenylpropanoid derivatives such as trans-cinnamic acid serve as precursors for assembly into suberin polyphenolics and polyaliphatics which in turn are modified, polymerized and form suberized layers at wounded sites [22]. Moreover, some polyphenolics derived from phenylpropanoids have been found to be toxic to phytopathogens [54]. Hence, the differential expression of the cutin monomer biosynthesis gene *CYP94A2* (XP_006351638.1) in the cutin, suberin, and wax biosynthesis pathways (Sot00073), the expression of genes in the phenylpropanoid biosynthesis pathways (Sot00940), as well as those in the flavonoid biosynthesis (Sot00941) observed in the current study are good indications of the clear genetic differentiation and differences in metabolic processes between HB and GM. Thus, genes of these metabolic processes may be good targets for future detailed functional studies. *CYP94A1*, a close relative of *CYP94A2* and a strict ω-hydroxylase that hydroxylates broad range chain fatty acids (10–18 chain), has been reported to be induced by abiotic stress [55] and to be involved in cutin synthesis and defense [56] whereas *CYP94A2* (XP_006351638.1) found up-regulated in GM and hydroxylating short-chain fatty acids (12–16 chain) was previously reported as being involved in signaling following biotic and abiotic stress [57]. *CYP94A2* up-regulation in the susceptible cultivar may be a response to the scab wounding by triggering cutin biosynthesis as an attempt to heal the wounded tissue through the activation of the jasmonic acid (JA) signalling pathway which requires the 16:3 and/or 18:3 fatty acids as precursors [58,59]. In line with this, the SA signalling that is an antagonist pathway to JA was down-regulated in GM, whilst the SA signaling (XP_015160896.1, XP_006346475.1), cell wall thickening genes related to N-Glycan (Sot00510) as well as the xyloglucan (XM_006348955.2) biosynthesis [60–62] were up-regulated in the resistant cultivar HB. These observations suggest an activated defense process involving SA signaling [25] and are consistent with previous findings reporting on the roles played by cell wall thickening in plant immunity [63]. Taken together, genes found differentially expressed in the early tuberization stage of the susceptible and resistant potato cultivars during infection [21] appeared to be also differentially expressed in the mature tubers of resistant and susceptible cultivars in the current study. This observation suggests that although RNA transcriptomic expression analyses at early stages of tuberization may help in understanding the cellular processes mounting the defense responses [9], it may not be essential for the determination of genes and pathways contributing to the resistance phenotype.

### Differential expression of resistance and resistance gene analogs in HB and GM

Disease and stress resistance genes were differentially expressed between GM and HB. By examining the onset of the scab infection process during tuberization in a scab susceptible and a relatively resistant potato cultivar, Dees et al. [21] also found a distinct DEG pattern between the cultivars in response to *S*. *turgidiscabies*. With the exception for thaxtomin A resistant 1 (Txr1) protein, a protein believed to facilitate the uptake and transport of thaxtomin A [64], the current transcriptomic dataset showed differentially expression of the GO terms and almost all transcripts reported by Dees et al. [21]. Our dataset did not show any differential expressed transcripts for *Txr1* probably because of its transient expression during the early infection process, its constitutive low transcript abundance, or its decay in the mature tubers used in this study. Whereas *Txr1* was not detected, transcripts of many disease resistance and stress-related tolerance genes including, putative disease resistance genes, *RGA4*, *RPP13*, *Mlo*, *Mpl* and thioredoxin 1 like protein (*Trx1*) previously reported by Dees et al. [21] were found differentially expressed (S2 and S3 Tables) along with isoforms of the chloroplastic *thioredoxin like 1–1*. Interestingly, the expression of the Putative late blight resistance protein was found to be up-regulated in HB when compared with GM through both RNAseq and qPCR transcriptomic profiling. Strikingly however, *RPP13* and *RGA4* were found up-regulated in GM. *RPP13* functions as a guard to plants against pathogens that contain an appropriate avirulence protein, and in contrast to other resistance proteins, it seems to work independently from the SA signaling pathway [65]. *RGA4* is reported as a defense protein mediating cell death [66]. With its scab necrotic phenotype and low SA signaling capacity, the high expressions of these two genes in GM seem to be an alternate signaling path used to heal the wounding. The *Mlo* gene family represent a typical class of susceptibility genes (S-genes) which genetic factors, when knockout, lead to recessive *mlo* resistance [67]. Two isoforms of *Mlo1* (XP_006344359.1 and XP_006351085.1) one of *Mlo8* (XP_006366655.1/XP_006366656.1), and one of *Mlo13* (XP_006358492.1) were more expressed in HB than in GM (S2 Table). Members of the *Mlo1* and *Mlo8* genes were also reported by Dees et al. [21] along with members of *Mlo4*, *Mlo5*, *Mlo6*, and *Mlo11* genes. Specific members of the *Mlo* gene family have been reported as powdery mildew (PM) susceptibility genes because their loss-of-function mutations lead to durable and broad-spectrum resistance [67,68]. In contrast to *Mlo1* and *Mlo8*, one isoform of *Mlo12* (XP_015169916.1) was more expressed in GM than in HB highlighting the diversity in the gene family. *Mlo* based-resistance has been reported in many other crops including pea [69] and tomato [70] and the negative regulation of vesicle-associated and actin-dependent defense pathways at the powdery mildew penetration site has been proposed as part of *Mlo* S-gene functions [71]. Moreover, the powdery mildew pathogen penetration is believed to be controlled by the secretory vesicle traffic, allowing the formation of cell wall appositions and papillae formation [72,73]. Here, based on the induction of cell biosynthetic genes in HB, cell wall apposition and strengthening also appeared to be a scab resistance mechanism in potato, and one may not rule out mutations in HB genes such as *Mlo* and functioning along with other cell wall-building and detoxification mechanisms. Indeed, the roles for all members of the *Mlo* gene family are still unknown, some of which with uncharacterized functions and/or others closely related genes to the unknown S-genes in plant-bacterial interactions may play a role in scab resistance in the resistant cultivars. Thus, *Mlo* gene family [74] may be a good target for deeper characterization and gene knockout through gene editing. Likewise, the *Mpl28* (XP_006367968.1, XP_006365602.1) and *Mpl34* (XP_006367717.2) were up-regulated in GM. The role of Mpl proteins in plant defense is well known [75] as is that of *Trx1* in cellular redox regulation and response to oxidative stress [76]. However, more specific and detailed studies in the potato-scab interactions are required to elucidate their exact roles in this pathosystem.

### Stronger induced-priming reactions in HB than GM

Along the genes described above, MPKs (such as *MPK3)* and serine threonine kinase *EDR1* have been reported to be involved in priming [23,29], and *EDR1* is suggested to mediate resistance through SA-inducible defense signaling of enzymes that mediate priming [23,29]. The current study showed a higher activation of these genes in HB than GM from the RNAseq dataset as well as a stronger time-dependent induction in HB from the greenhouse experiment, suggesting an “activated priming state” in HB. This assumption is also substantiated by the fact that subtilisin-like protease, a gene whose role in plant-pathogen recognition and immune priming is known in plants [77] was highly (6 folds) down-regulated in GM, as were also *MPK3* and *EDR1*, in comparison with HB. Nonetheless, as a field trial, it cannot be ruled out that the field priming events might have also involved plant interactions with other microorganisms [26] in addition to interactions with scab evidenced in the current study. Indeed, it is now well known and accepted that priming is an intrinsic part of induced resistance during which the plant takes defensive measures against the potential attackers while also preparing its defensive system for faster and/or stronger reactions to future challenges [24,30]. It is also accepted that plants are at least partly prime-induced through interactions with both biotic and abiotic environment under field condition [26]. In the context of our study, certified seed potatoes were obtained from conventional certified production system through 6–7 years of field-growing and testing from the nuclear seed stage, and hence, were previously exposed to non-pathogenic, pathogenic or endophytic microorganisms, both of which can mediate priming and which effects can be retained transgenerationally [27,30] in the daughter tubers. Moreover, the current study used a field with an artificial soil-built-in microbiome communities including a high scab inoculum. It is therefore reasonable to assume that some of these communities (endophytes, non-pathogenic and pathogenic) might have first attacked the root system of both cultivars prior to the tuberization, hence inducing a prior cellular priming, and then by scab during and after the tuberization to initiate tuber’ cellular priming and memorization through the PAM/MAMP-triggered or effector-triggered immunity [53]. Although it is not possible to ascertain directly the specific priming organisms prior to scab interactions in the field conditions described, it is without doubt that both cultivars were submitted to scab pressure as shown by the scab disease data reported here (from both the field and greenhouse experiments) and by the time course gene expression and priming signaling data. Thus, the plants appeared to have been primed as evidenced by HB’s strong gene induction and disease resistance responses in a time-dependent manner during tuber development. Furthermore, the PCA analysis correlated the gene expressions of *EDR1* and *Subtilisin* to the disease symptoms, suggesting potential active roles for these genes in the defense mechanism. Although a PCA analysis *per se* can not accurately explain the interaction of the studied genes, activation of these priming genes [28–31] was established in the current study. Nonetheless, a more detailed study is required to assess the structural gene organization in HB and GM as well as the potential interactions between genes.

### Differential activation of signalling and transcriptional regulation in HB and GM

Two key mechanisms, physical and chemical, have been suggested in scab resistance [9,63]. In the current study, genes associated with cell wall formation and strengthening including N-glucan and lignin, as well as those involved in early defense signalling such as LRR receptor-like kinase, *MAPK8* and *PTI4*, and in detoxification (terpenoid, sesquiterpenoid and triterpenoid, phenylpropanoid, flavonoid, tropinone, piperidine and pyridine) were more expressed in HB than in GM. Interestingly, brassinosteroid biosynthesis in the terpenoid pathways was altered in GM. The role of brassinosteroid-mediated cell wall remodeling under stress has been reported [78] and the data reported here tend to support the structural differences of the tuber’s skin cell wall, thus impacting on the scab symptoms between the two cultivars. LRR subclass that includes the receptor-like kinase superfamily has been reported as acting in broad-spectrum, elicitor-initiated defense signaling responses as well as dominant resistance R genes in race-specific pathogen defense [79]. The differential expression of LRR receptor kinases EFR and EFI 2 isoforms further underlies the genetic and metabolic differences between the two cultivars, and isoform EFI 2 may likely be involved in scab resistance [79]. As far as MAPK kinases and PTI4 are concerned, MAPK kinases have been reported to be involved in mediating ROS signaling during abiotic and biotic stress [80] whereas *PTI4*, which is part of the pathogen-associated molecular patterns (PAMPs)-triggered immunity (PTI) motif [63] and being a pathogenesis-related gene transcriptional activator, was found down-regulated in GM. *PTI4* has been shown associated with HR response and induction of defense-related gene response to *Avr* factors of bacterial secretion system [63,81,82]. The up-regulation of *MAPK8* and *PTI4* genes in HB than in GM suggests that the activation of their transcript in the resistant cultivar may not be necessarily dependent on active pathogenesis as occurred during infection at the early stage of tuberization.

In our study, the transcription factor *bHLH* was 5 fold more expressed in HB than in GM. Moreover, no transcripts for *MYB21* was found in any of the replicated samples from GM compared with HB, where the gene was highly expressed in all replicates. A similar correlation between the expression of transcription factors and scab resistance was previously reported by [83]. The roles of *MYb* and *bHLH* transcription factors in secondary metabolites are well known [84] and their coregulated differential expression with that of other genes involved in biosynthesis of secondary metabolites such as flavonoids, terpenoid, sesquiterpenoid and triterpenoid, phenylpropanoid, flavonoid, tropinone, piperidine and pyridine, may be an indication of their pivotal roles in scab resistance through the production of toxic activities by these metabolites. Moreover, the differential expression of the WRKY transcription factors observed in the current study was noticeable and congruent with the report by Enciso-rodriguez et al. [85] who associated a SNP variant in found WRKY with common scab resistance. However, more studies comparing the gene structural organization of *MAPK8*, *PTI4*, *WRKY*, *Myb21* and *bHLH* in HB and GM would help determining the potential allelic variants that may explain the transcriptional difference and guide in designing approaches for targeted editing of these genes in scab-susceptible cultivars.

### Conclusion

In conclusion, this study allowed us to show a clear and contrasting differential gene expression between resistant and susceptible potato cultivars to common scab and, thus, to demonstrate that tissue from mature potato tuber can be used to discriminate cultivars for scab resistance using transcriptional expression profiles in the absence of active pathogenesis. It also showed that HB had mounted an ability to sense and prime itself for persistent response to scab disease. Taken together, some of the key genes reported here and some of those reported by Dees et al. [21], pave the way for further functional characterizations in the potato-scab pathosystem and can be used as markers for screening potato breeding lines at any developmental stage for scab resistance without a need for a whole RNA sequencing transcriptomic analysis.

## Supporting information

S1 Table List of primers used for priming evaluation using qPCR. (XLSX)

S2 Table List of differentially expressed transcripts. (XLSX)

S3 Table List of the 1,064 genes differentially expressed in potato cultivars Green Mountain and Hindenburg. A total of 501 genes were down-regulated in the susceptible cultivar Green Mountain whereas 563 were up-regulated in the resistant cultivar Hindenburg.
(XLSX)

S1 Fig Venn diagram representing the number of A) spliced transcript variants which absolute expression fold change (|fc|) was ≥2 observed in at least two biological replicated samples of the scab susceptible cultivar Green Mountain and resistant cultivar Hindenburg and B) their coding proteins. Only protein coding spliced variants were considered and reported.
(TIF)

S2 Fig Coordinated down-regulation of genes in the terpenoid and brassinosteroid biosynthetic pathway of the scab susceptible cultivar Green Mountain when compared with the resistant cultivar Hindenburg. Purple and light blue, down-regulated genes in GM compared with HB; Yellow, unchanged gene expression in the two cultivars. Significant pathway module is marked with a red star.
(TIFF)

S3 Fig Time-course gene expression studies of signaling and priming-associated genes (*MPK3*, *EDR1*, *Subtilisin*) and *Mlo* genes in tuber samples in four treatments, as performed by qPCR in susceptible cultivar Green Mountain (GM) and resistant cultivar Hindenburg (HB). Vertical bars represent standard deviation from the means of three biological replications from each treatment. naClean, non-autoclaved soil planted with certified clean seed; naScab, non-autoclaved soil planted with scab-infected seed from the 2018 infected field; acClean, autoclaved soil planted with certified clean seed; acInoScab, autoclaved soil inoculated with scab inoculum and planted with scab-infected seed from the 2018 infected field.
(TIFF)

S4 Fig Principal component analysis (PCA) plot showing association between time-course of signaling and priming-associated gene expressions at time point 1 and 2 with scab disease symptom rating. Only variations explained by PCA score 1 and 3 are shown.
(TIFF)
