# Supplementary figures and images for: Comparative transcriptome expression analysis in susceptible and resistant potato (Solanum tuberosum) cultivars to common scab (Streptomyces scabies) revealed immune priming responses in the incompatible interaction
Source: PLoS One. 2020 Jul 16;15(7):e0235018. doi: 10.1371/journal.pone.0235018 (PMC7365407; doi:10.1371/journal.pone.0235018)

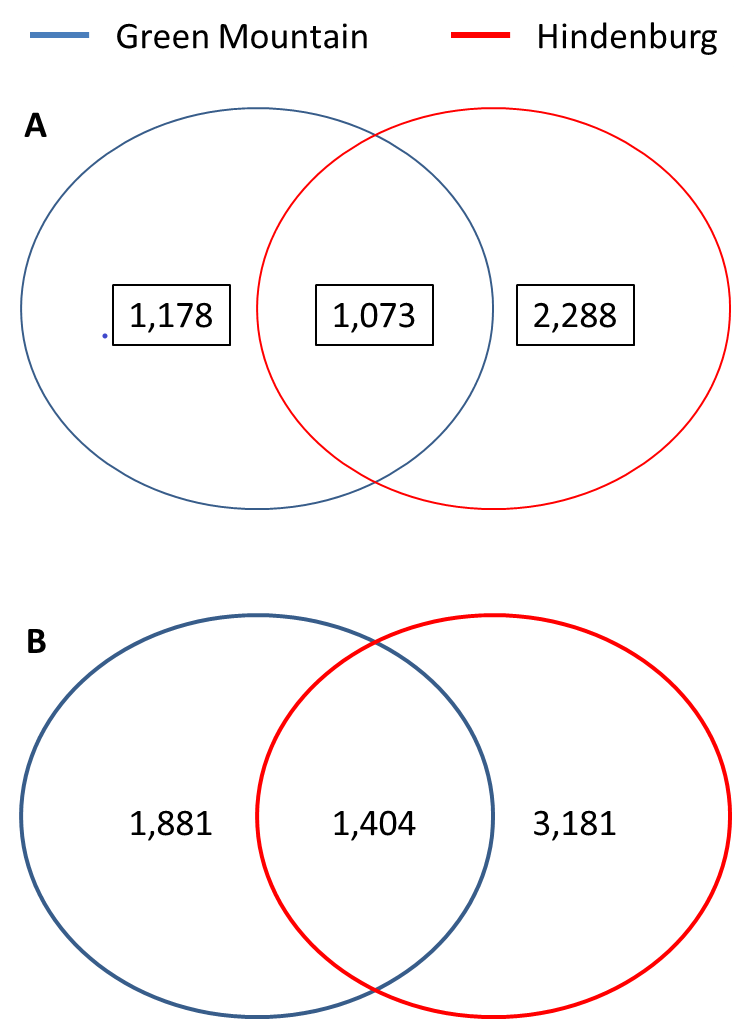

Supplement: S1 Fig — Only protein coding spliced variants were considered and reported. (TIF) [file pone.0235018.s004.tif]

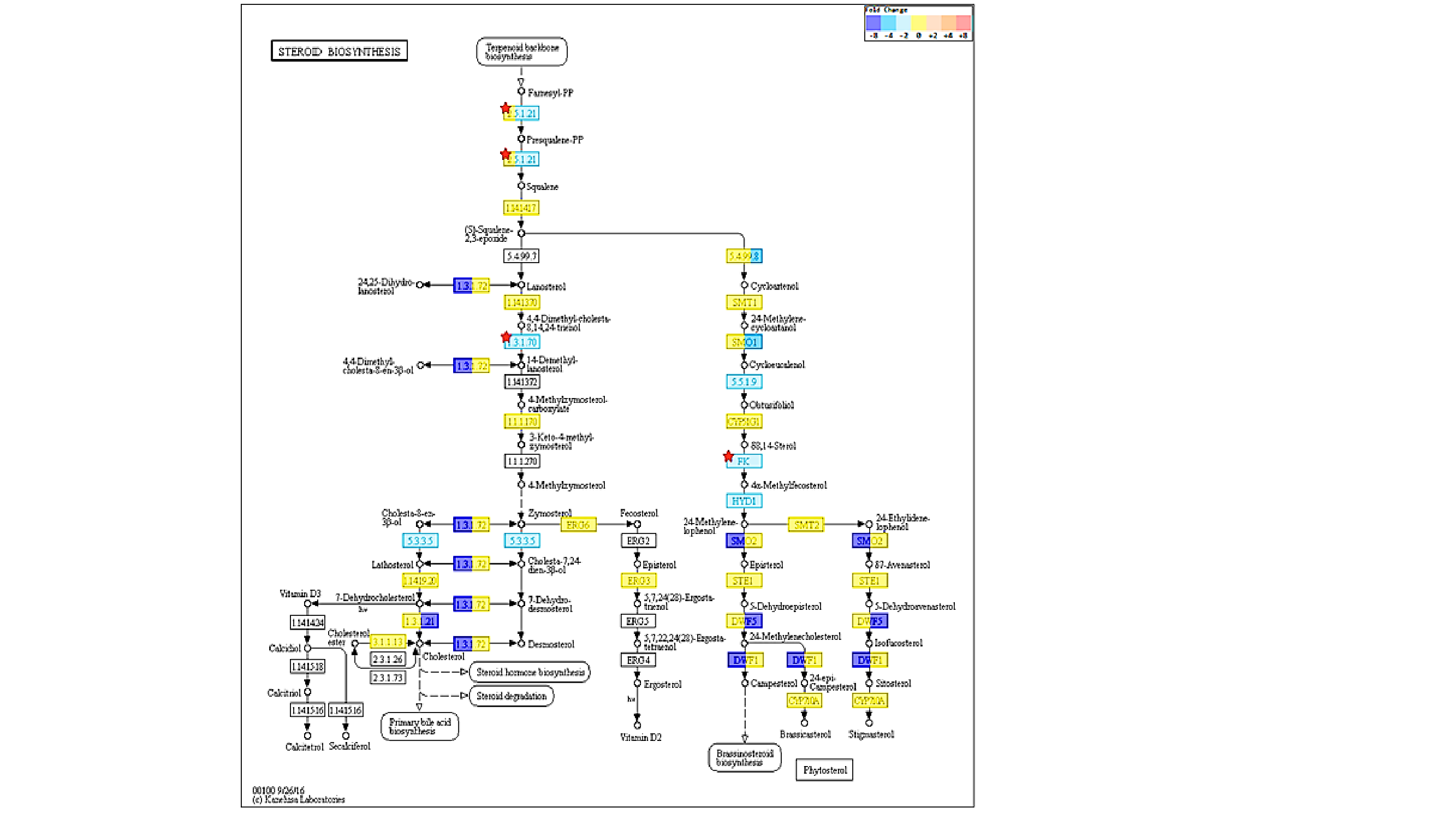

Supplement: S2 Fig — Purple and light blue, down-regulated genes in GM compared with HB; Yellow, unchanged gene expression in the two cultivars. Significant pathway module is marked with a red star. (TIFF) [file pone.0235018.s005.tiff]

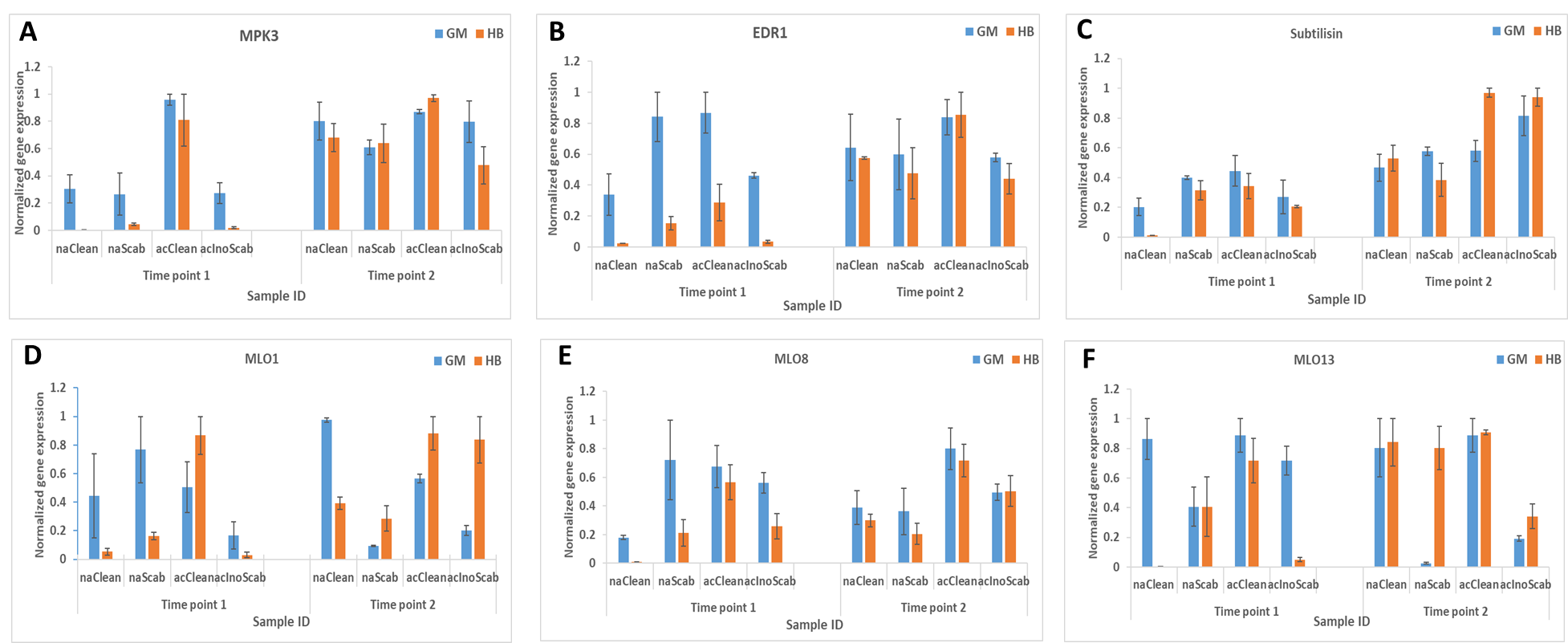

Supplement: S3 Fig — Vertical bars represent standard deviation from the means of three biological replications from each treatment. naClean, non-autoclaved soil planted with certified clean seed; naScab, non-autoclaved soil planted with scab-infected seed from the 2018 infected field; acClean, autoclaved soil planted with certified clean seed; acInoScab, autoclaved soil inoculated with scab inoculum and planted with scab-infected seed from the 2018 infected field. (TIFF) [file pone.0235018.s006.tiff]

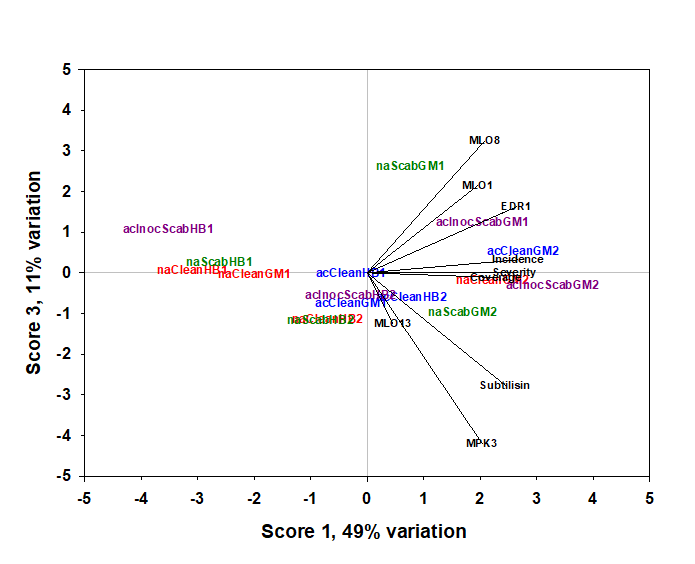

Supplement: S4 Fig — Only variations explained by PCA score 1 and 3 are shown. (TIFF) [file pone.0235018.s007.tiff]
